# Supplementary material for: Antenatal, intrapartum and infant azithromycin to prevent stillbirths and infant deaths: study protocol for SANTE, a 2×2 factorial randomised controlled trial in Mali
Source: BMJ Open. 2023 Aug 30;13(8):e067581. doi: 10.1136/bmjopen-2022-067581 (PMC10471877; doi:10.1136/bmjopen-2022-067581)
Supplement: Supplementary data [file bmjopen-2022-067581supp001.pdf]

SANTE Protocol Manuscript – SUPPLEMENTAL APPENDIX – December 21, 2022

Supplemental Online Appendix.

Supplemental Table 1. World Health Organization Trial Registration Data Set

**Primary Registry and Trial Identifying Number**	ClinicalTrials.gov; NCT03909737
**Date of Registration in Primary Registry**	April 10, 2019
**Secondary Identifying Numbers**	
**Source(s) of Monetary or Material Support**	Bill & Melinda Gates Foundation
**Primary Sponsor**	University of Maryland, Baltimore
**Secondary Sponsor(s)**	n/a
**Contact for Public Queries**	Amanda Driscoll PhD<br>adriscoll@som.umaryland.edu
**Contact for Scientific Queries**	Karen Kotloff MD<br>Karen Kotloff kkotloff@som.umaryland.edu
**Public Title**	The SANTE trial
**Scientific Title**	Sauver avec l'Azithromycine en Traitant les Femmes Enceintes et les Enfants / Save with Azithromycin by Treating Pregnant Women and Children (SANTE)
**Countries of Recruitment**	Mali
**Health conditions or problems studied**	The burden of stillbirths and infant mortality remains high in low-income settings. Prophylactic azithromycin delivered in pregnancy and in infancy has shown promise as a potential intervention to improve birth outcomes and infant survival.
**Interventions**	Pregnant women: a single 2g dose of oral azithromycin (or placebo) delivered in the second and third trimesters of pregnancy, and at delivery<br>Infants: a single 20mg/kg dose of oral azithromycin (or placebo) delivered concurrently with the first and third doses of diphtheria, tetanus, pertussis containing vaccine.
**Key Inclusion and Inclusion Criteria**	At enrollment, pregnant women must be ≥14 weeks gestation and must not be in active labor. Infant participants must be ≥6 weeks of age at enrollment and/or the time of the first dose. Participants must plan to reside in the study area for at least 6 months after the infant is born. Participants who are being treated with azithromycin or another macrolide antibiotic for a medical condition are excluded from enrollment.
**Study Type**	Individually randomized 2x2 factorial placebo-controlled trial
**Date of First Enrollment**	September 2020
**Sample size**	49,600 mother-infant pairs, plus an additional 12,000 infants

Driscoll AJ, *et al. BMJ Open* 2023; 13:e067581. doi: 10.1136/bmjopen-2022-067581

SANTE Protocol Manuscript – SUPPLEMENTAL APPENDIX – December 21, 2022

**Recruitment Status**	Recruiting
**Primary Outcome(s)**	Primary outcome 1A: a composite of stillbirths and infant mortality within the first 6-12 months of life<br>Primary outcome 1B: infant mortality between the time of first does through 6-12 months of life
**Key Secondary Outcomes**	Low birth weight (<2500g), neonatal death (0-<28 days), and a composite outcome of maternal hospitalizations and mortality through six weeks postpartum. The cost effectiveness of the interventions will also be estimated as a secondary aim.
**Ethics Review**	This trial was approved by the Institutional Review Board at the University of Maryland School of Medicine (Protocol #HP-00084242) and the Faculté de Médecine et d'Odonto-Stomatologie in Mali
**Completion Date**	n/a
**Summary Results**	n/a
**IPD Sharing Statement**	Access to trial IPD may be provided to qualified researchers upon request and following review and approval of a research proposal and execution of a Data Sharing Agreement (DSA).

Supplemental Table 2. Characteristics of the Study Settings

	Sikasso Region (SANTE study area)	Comparator Indicators		
		Mali, national level indicators	African Region	Global
Under-five mortality rate per 1000 live births	111[1]	101[1]	76[2]	38[2]
Infant mortality rate per 1000 live births	67[1]	54[1]	52[2]	28[2]
Neonatal mortality rate per 1000 live births	42[1]	33[1]	27[2]	17[2]

SANTE Protocol Manuscript – SUPPLEMENTAL APPENDIX – December 21, 2022

Perinatal deaths per 1000 births (stillbirths plus early neonatal deaths w/in 7 days)[2]	45[3]	38[3]		
Stillbirths per 1000 births	7[3]	12[3] / 19.7[4]	21.7[4]	13.9[4]
Proportion of pregnancies where antenatal care was received by a trained provider	76.1%[5]	79.5%[5]		
Proportion of pregnant women that received 3+ doses of IPTp	26.8%[6]	28.3%[6]	35%[7]	
Proportion of births that were facility-based	71.0%[5]	66.8%[5]		
Proportion of births attended by a skilled provider	70.8%[5]	67.3%[5]		
Proportion of children 12-35 months of age who received DTP-1	85.7%[8]	82.1%[8]		
Proportion of children 12-35 months of age who received DTP-3	69.4%[8]	70.7%[8]		

[1]From Mali 2018 DHS Report, Tables 8.1 and 8.2

[2] 2019 data, from Levels & Trends in Child Mortality. Report 2020. Estimates developed by the UN Inter-agency Group for Child Mortality Estimation.

[3]From Mali 2018 DHS Report, Table 8.4. Stillbirths calculated as the difference between perinatal and early neonatal deaths, and defined as fetal deaths occurring at 7 months or more of pregnancy

[4] 2019 estimates from: Global, regional, and national estimates and trends in stillbirths from 2000 to 2019: a systematic assessment. 2021. Lancet 398:772-785. Stillbirth defined as fetal death at 28 weeks gestation or more.

[5]From Mali 2018 DHS Report, Tables 9.1, 9.5 and 9.6 – Trained providers defined as doctors, nurses, and midwives.

[6]IPTp= Intermittent preventive treatment for malaria during pregnancy. From Mali 2018 DHS Report, Table 12.9

[7]2019 estimate from World Malaria Report, 2021. World Health Organization. https://www.who.int/teams/global-malaria-programme/reports/world-malaria-report-2021

[8] DTP=Diphtheria, tetanus and pertussis containing vaccine. From Mali 2018 DHS Report, Table 10.3

SANTE Protocol Manuscript – SUPPLEMENTAL APPENDIX – December 21, 2022

Supplemental Table 3. Gestational Age Algorithm*

**For participants with only fundal health (FH) measurement available:**	
If FH measurement ≥16 cm or ≥35 cm	Assume cm FH = weeks gestational age
If FH measurement <16 cm	Assume GA= 14-15 weeks
If FH measurement >35 cm	Assume GA>35 weeks
**For participants with date of last menstrual period (LMP date) available:**	
If no FH or ultrasound dating (US) available	Use LMP date
If no US available and difference between LMP date and FH is +/- 14 days	Use LMP date
If no US available and difference between LMP date and FH is > 14 days	Use FH
If US is available:	
Earliest US at <9 weeks	
Within +/- 5 days of LMP date	Use LMP date
> 5 days different from LMP date	Use US
Earliest US at 9 to <16 weeks	
Within +/- 7 days of LMP date	Use LMP date
> 7 days different from LMP date	Use US
Earliest US at 16 to <22 weeks	
Within +/- 10 days of LMP date	Use LMP date
> 10 days different from LMP date	Use US
Earliest US at 22 to <28 weeks	
Within +/- 14 days of LMP date	Use LMP date
> 14 days different from LMP date	Use US
Earliest US at ≥28 weeks	
Within +/- 21 days of LMP date	Use LMP date
> 21 days different from LMP date	Use US
**For participants without FH, LMP date, or US**	Estimated GA is missing

*Adapted with permission from the Azithromycin-Prevention in Labor Use Study (A-PLUS) investigators https://clinicaltrials.gov/ct2/show/NCT03871491

SANTE Protocol Manuscript – SUPPLEMENTAL APPENDIX – December 21, 2022

Supplemental Figure 1. Infantile Hypertrophic Pyloric Stenosis Reporting Algorithm

Asked at each infant visit: Has the infant had forceful vomiting after feeding for 2 or more days, with vomit the color of breastmilk?

↓

If yes, complete Adverse Event of Special Interest Form, shown below

**1.** Date of notification:	___ ___/___ ___ ___/___ ___ ___ ___ (dd/MMM/yyyy)
**2.** Date symptoms began	___ ___ ___ ___ Year (*enter 9999 if unknown*)<br>___ ___ ___ Month (*enter XXX if unknown*)<br>___ ___ Day (*enter 99 if unknown*)
**3.** Are the symptoms ongoing?	☐YES (*skip to 4*)<br>☐NO<br>☐Unknown (*skip to 4*)
**3a**. (If NO to Q3) End date of symptoms	___ ___ ___ ___ Year (*enter 9999 if unknown*)<br>___ ___ ___ Month (*enter XXX if unknown*)<br>___ ___ Day (*enter 99 if unknown*)
**4.** Date of the last dose of study drug:*	___ ___/___ ___ ___/___ ___ ___ ___<br>(dd/MMM/yyyy)
**Event information**	
**5.** Age of the infant participant*	___ ___ weeks
**6.** Sex of the infant participant*	☐Male ☐Female ☐ Indeterminate
**7.** How many consecutive days did the vomiting last?	________ Days
**8.** Does the infant participant seem to be hungry most of the day? (*select one option*)	☐ YES ☐ NO ☐ Unknown
**9.** Has the infant participant had diarrhea?	☐ YES ☐ NO ☐ Unknown
**10.** Did the parent/caregiver seek medical care for this vomiting episode? (*If NO, refer for a medical evaluation if the symptoms are ongoing*)	☐ YES ☐ NO ☐ Unknown
**10a**. (If YES to Q10) What was the diagnosis? (*select one option*)	☐Pyloric stenosis<br>☐Gastroenteritis<br>☐Other, specify: __________<br>☐ Unknown
**10b**. (If YES to Q10) How was the diagnosis made? (*choose all options that apply*)	☐ Clinical exam (feeling a mass)<br>☐ Ultrasound<br>☐ Surgery<br>☐ Other, specify ________________<br>☐ Unknown
**10c.** (If YES to Q10) What care was provided? (*choose all options that apply*)	☐ Medicine<br>☐ Surgery<br>☐ None<br>☐ Unknown
**11.** Pyloric stenosis diagnosis*	☐ Possible (Forceful vomiting of 2 days or more color of breastmilk)<br>☐ Probable (Possible, with palpitation of a mass) (*Complete SAE form*)<br>☐ Definite (Sonogram, ultrasound, or surgical diagnosis) (*Complete SAE form*)

Driscoll AJ, *et al. BMJ Open* 2023; 13:e067581. doi: 10.1136/bmjopen-2022-067581

SANTE Protocol Manuscript – SUPPLEMENTAL APPENDIX – December 21, 2022

Randomization Procedures

Randomization for the main study will be performed via stratified permuted-block design.

For the mother-infant cohort:

- Randomization of the mother-infant pairs will be stratified by facility and will occur at the enrollment ANC visit
- Since the table of randomization numbers and corresponding treatment assignments will be generated in advance, extra randomization numbers will be generated for each (facility) as we do not know in advance how many women will be randomized within each stratum
- Within each stratum, randomization to treatment groups A1, A2, B1 and B2 will be performed using a permuted block design with randomly varying block sizes of 8 and 16
  - A total of 4,992 treatment assignments will be generated in each stratum and there will be a total of 416 blocks – 208 of size 8 and 208 of size 16
- Within each block, 25% of the treatment assignments will be allocated to each treatment (A1, A2, B1, B2)
- Mother and infant treatment assignments will each be allocated two bins; thus, there will be four bins (two for active and two for placebo) for mother treatments and four bins (two for active and two for placebo) for infant treatments
- Bins will be labeled with letters, as in the following example, for a total of 4*2 (8) different randomization assignments:
  - **Mother (active) – bins A and B;**
  - **Mother (placebo) – bins C and D;**
  - **Infant (active) – bins E and F;**
  - **Infant (placebo) – bins G and H;**
- Bins will be assigned to each mother/infant treatment assignment in equal proportion. For example, half of the mother (active) randomization assignments will be bin A and the other half will be bin B.
- Each randomization assignment will be associated with one bin.

For the infant-only cohort:

- Randomization will be stratified by facility
- Since the table of randomization numbers and corresponding treatment assignments will be generated in advance, extra randomization numbers will be generated for each stratum as we do not know in advance how many infants will be randomized within each stratum
- Within each stratum, randomization to treatment groups 1 and 2 will be performed using a permuted block design with randomly varying block sizes of 4 and 8
  - A total of 1800 treatment assignments will be generated in each stratum and there will be a total of 300 blocks –150 of size 4 and 150 of size 8

- Within each block 50% of the treatment assignments will be allocated to each treatment (1 and 2)
- Infant treatment assignments will each be allocated two bins; thus, there will be four bins (two for active and two for placebo)
- Bins will be labeled with letters, as follows, for a total of 2*2 (4) different randomization assignments:
  - **Infant (active) – bins E and F**
  - **Infant (placebo) – bins G and H**

Bins will be assigned to each infant treatment assignment in equal proportion

Supplemental Table 4. Protocol Modifications

Protocol version	Date	Major modifications
1.0	14Jan2019	Original submission
2.0	13Mar2019	-Added descriptions of ancillary studies<br>-Added pilot acceptability testing<br>-Added procedures for obtaining consent electronically<br>-Increased overall enrollment number
3.0	19Oct2019	-No major modifications; administrative clarifications only
4.0	19Nov2019	-No major modifications; administrative clarifications only
5.0	20Mar2020	-Gestational age requirement increased from 12 weeks to 14 weeks<br>-Added requirement for infants to be minimum 6 weeks of age for enrollment in infant-only cohort<br>-Removed pilot acceptability testing<br>-Clarified description of consenting procedures
6.0	12Mar2021	-Clarified infant dosing procedures to allow study medication dosing despite pentavalent vaccination stockouts, or to allow dosing if the infant has received vaccine at a non-study facility<br>-Excluded stillbirths from SAE reporting; added harm analysis for stillbirths<br>-Added ancillary studies
7.0	24Jun2021	-Allowed contacts between formal visits to assess health and vital status<br>-Exclude infant deaths from SAE reporting; added harm analysis for infant deaths
8.0	15Sep2021	No major changes, administrative clarifications
9.0	19May2022	Collection of nasopharyngeal swabs for ancillary studies changed to nasal swabs
10	26Oct2022	-Removal of urban mother-infant cohort<br>-Removal of ancillary studies that will not be enrolled<br>-Updated sample size calculations and target sample sizes

		-Excluded unplanned caesarean sections and obstetric emergencies from SAE reporting; added harm analysis for caesarean sections and obstetric emergencies
