# Supplementary material for: Antenatal, intrapartum and infant azithromycin to prevent stillbirths and infant deaths: study protocol for SANTE, a 2×2 factorial randomised controlled trial in Mali
Source: BMJ Open. 2023 Aug 30;13(8):e067581. doi: 10.1136/bmjopen-2022-067581 (PMC10471877; doi:10.1136/bmjopen-2022-067581)
Supplement: Author's manuscript [file bmjopen-2022-067581.draft_revisions.pdf]

BMJ Open is committed to open peer review. As part of this commitment we make the peer review history of every article we publish publicly available.

When an article is published we post the peer reviewers' comments and the authors' responses online. We also post the versions of the paper that were used during peer review. These are the versions that the peer review comments apply to.

The versions of the paper that follow are the versions that were submitted during the peer review process. They are not the versions of record or the final published versions. They should not be cited or distributed as the published version of this manuscript.

BMJ Open is an open access journal and the full, final, typeset and author-corrected version of record of the manuscript is available on our site with no access controls, subscription charges or pay-per-view fees (http://bmjopen.bmj.com).

If you have any questions on BMJ Open's open peer review process please email info.bmjopen@bmj.com

BMJ Open

Antenatal, intrapartum, and infant azithromycin to prevent stillbirths and infant deaths: study protocol for SANTE, a 2x2 factorial randomized controlled trial in Mali

Journal:	*BMJ Open*
Manuscript ID	bmjopen-2022-067581
Article Type:	Protocol
Date Submitted by the Author:	22-Aug-2022
Complete List of Authors:	Driscoll, Amanda; University of Maryland School of Medicine, Center for Vaccine Development and Global Health<br>Haidara, Fadima; Center for Vaccine Development–Mali<br>Tapia, Milagritos; University of Maryland School of Medicine, Center for Vaccine Development and Global Health<br>Deichsel, Emily L.; University of Maryland School of Medicine, Center for Vaccine Development and Global Health<br>Samake, Ousmane S.; Centre pour le Développement des Vaccins<br>Bocoum, Tiecoura; Centre pour le Développement des Vaccins<br>Bailey, Jason A.; University of Maryland School of Medicine, Center for Vaccine Development and Global Health<br>Fitzpatrick, Meagan ; University of Maryland School of Medicine, Center for Vaccine Development and Global Health<br>Goldenberg, Robert ; Columbia University School of Medicine, Obstetrics and Gynecology<br>Kodio, Mamoudou; Centre pour le Développement des Vaccins<br>Moulton, Lawrence H.; Johns Hopkins Bloomberg School of Public Health<br>Nasrin, Dilruba; University of Maryland School of Medicine, Department of Medicine<br>Onwuchekwa, Uma; Centre pour le Développement des Vaccins<br>Shaffer, Allison M.; University of Maryland School of Medicine, Center for Vaccine Development and Global Health<br>Sow, Samba O.; Center for Vaccine Development; Centre pour le Développement des Vaccins<br>Kotloff , Karen ; University of Maryland School of Medicine, Center for Vaccine Development and Global Health
Keywords:	INFECTIOUS DISEASES, PERINATOLOGY, PAEDIATRICS, EPIDEMIOLOGY

SCHOLARONE™
Manuscripts

Antenatal, intrapartum, and infant azithromycin to prevent stillbirths and infant deaths: study protocol for SANTE, a 2x2 factorial randomized controlled trial in Mali

Amanda J. Driscoll[1]*, Fadima C. Haidara[2]*, Milagritos D. Tapia[1,5], Emily L. Deichsel[1], Ousmane S. Samake[2], Tiecoura Bocoum[2], Jason A. Bailey[1], Meagan C. Fitzpatrick[5], Robert L. Goldenberg[4], Mamoudou Kodio[2], Lawrence H. Moulton[3], Dilruba Nasrin[5], Uma Onwuchekwa[2], Allison M. Shaffer[1], Samba O. Sow[2]**, Karen L. Kotloff[1,5]**

[1]Department of Pediatrics, Center for Vaccine Development and Global Health, University of Maryland School of Medicine, Baltimore.

[2]Centre pour le Développement des Vaccins, Bamako, Mali.

[3]Department of International Health, Johns Hopkins Bloomberg School of Public Health, Baltimore, MD, USA.

[4]Department of Obstetrics and Gynecology, Columbia University School of Medicine, New York, NY, USA.

[5]Department of Medicine, Center for Vaccine Development and Global Health, University of Maryland School of Medicine, Baltimore

*Amanda J. Driscoll and Fadima C. Haidara contributed equally to this work.

**Karen L. Kotloff and Samba O. Sow contributed equally to this work.

Corresponding Author:

Amanda J. Driscoll, MHS PhD

Center for Vaccine Development and Global Health

University of Maryland, Baltimore

685 W. Baltimore St.

Baltimore, MD 21201

Telephone (410) 706-5328

Email: adriscoll@som.umaryland.edu

Abstract

Introduction Prophylactic azithromycin has been shown to improve birthweight and gestational age at birth when administered antenatally, to reduce the incidence of neonatal infections when administered

For peer review only - http://bmjopen.bmj.com/site/about/guidelines.xhtml

intrapartum, and to improve survival when administered in infancy. Questions remain regarding whether azithromycin can prevent stillbirths, and regarding the optimal strategy for the delivery of azithromycin in infancy.

Methods and analysis *Sauver avec l'Azithromycine en Traitant les Femmes Enceintes et les Enfants* (SANTE) is a 2x2 factorial, individually randomized, placebo-controlled, double-masked trial in rural Mali. The primary aims are: (1A) to assess the efficacy of antenatal and intrapartum azithromycin on a composite outcome of stillbirths and infant mortality through 6-12 months, and (1B) to assess the efficacy of azithromycin administered concurrently with the first and third doses of Pentavalent vaccines (Penta-1/3) on infant mortality through 6-12 months. Pregnant participants (n=33,600) seeking antenatal care at ≥14 weeks gestation are randomized 1:1 to receive two antenatal doses and one intrapartum dose of 2g azithromycin or placebo. Their infants, and an additional cohort of 8,500 infants enrolled at the Penta-1 vaccination visit, are randomized 1:1 to receive 20mg/kg azithromycin or placebo at the Penta-1 and Penta-3 vaccination visits.

Ethics and dissemination This trial was approved by the Institutional Review Board at the University of Maryland School of Medicine (Protocol #HP-00084242) and the Faculté de Médecine et d'Odonto-Stomatologie in Mali. The findings of this trial will be published in open access peer-reviewed journals.

Trial registration number ClinicalTrials.gov number: NCT03909737; pre-results

https://clinicaltrials.gov/ct2/show/NCT03909737

Keywords: infectious diseases, epidemiology, perinatology, clinical trials

ARTICLE SUMMARY

STRENGTHS AND LIMITATIONS OF THIS STUDY

(5 short bullet points, no longer than one sentence each, that relate specifically to the methods)

- The trial is conducted in a low-resource setting with high rates of stillbirth and infant mortality
- The intervention is delivered through the public healthcare system, relying on the existing infrastructure for routine primary care
- The trial assesses multiple timepoints for the administration of azithromycin to prevent infant mortality (antenatal, intrapartum, and infant)
- The trial includes ancillary studies to assess antimicrobial resistance and to investigate potential mechanisms of action of azithromycin in subsets of participants
- The trial is unable to reliably determine individual causes of stillbirth and infant death

For peer review only - http://bmjopen.bmj.com/site/about/guidelines.xhtml

INTRODUCTION

Despite significant improvements in child survival over the past two decades, global rates of stillbirth and infant mortality remain unacceptably high. Of the 5.2 million under-five deaths in 2019, 3.9 million (75%) occurred within the first 12 months of life and 2.4 million (45%) within the first month.(1) In sub-Saharan Africa, where more than half of global under-five deaths occur, approximately 60% of child mortality is estimated to be due to preventable infectious causes.(2) Sub-Saharan Africa also bears the highest burden of stillbirths, accounting for only 27% of total births but 42% of the estimated 2 million global stillbirths in 2019.(3) The causes of stillbirths in low- and middle-income countries (LMICs) are largely unexplained, owing to insufficient data and the failure to adopt a standardized classification system for perinatal cause of death.(4-6) Malaria and syphilis are the most recognized infectious causes of stillbirth, but additional research is needed to further elucidate the role of maternal infection and the contributions of other pathogens.(7)

An effective new strategy to prevent stillbirths and infant deaths that could be incorporated into routine primary healthcare services would be a major achievement in global health. One promising intervention to improve child survival is the prophylactic administration of the antibiotic azithromycin, a second-generation broad-spectrum macrolide that is used in mass drug administration (MDA) campaigns to control trachoma.(8) Current World Health Organization (WHO) guidelines recommend consideration of biannual MDA-azithromycin for infants aged 1-11 months to improve survival in high mortality settings.(9) These guidelines are based on data from three randomized controlled trials (RCTs), two of which found a statistically significant reduction in all-cause mortality among children randomized to receive azithromycin.(10-12) There are insufficient data to support global policy recommendations for prophylactic azithromycin in pregnancy, although it has also been shown to reduce the incidence of preterm birth and low birth weight when administered antenatally, and to prevent neonatal infections when administered intrapartum.(13, 14) Further evidence is needed to understand (1) whether antenatal and intrapartum azithromycin can prevent stillbirths and early infant deaths, (2) the optimal method and timing of azithromycin administration in infancy to prevent mortality, (3) whether the survival benefit observed in two randomized MDA-azithromycin trials can be replicated in other settings.

Here, we describe the protocol for an individually randomized, placebo-controlled,2x2 factorial trial to determine (1) the effect of antenatal and intrapartum azithromycin on stillbirths and infant mortality and, (2) the effect of azithromycin on infant mortality when delivered with the first and third doses of

For peer review only - http://bmjopen.bmj.com/site/about/guidelines.xhtml

diphtheria, tetanus, pertussis, hepatitis, and Hib-containing (i.e., pentavalent) vaccine. The trial will take place in Mali, a low-income West African country with high stillbirth, neonatal, and infant mortality.

METHODS AND ANALYSIS

Study Design, Objectives and Hypotheses

Sauver avec l'Azithromycine en Traitant les Femmes Enceintes et les Enfants (SANTE) is an individually randomized, placebo-controlled, double-masked trial. Using a 2x2 factorial design with four treatment arms, SANTE will simultaneously assess co-primary aim 1A: whether azithromycin administered at routine antenatal care (ANC) visits (second and/or third trimester) and at delivery reduces a composite outcome of stillbirth and infant mortality, and co-primary aim 1B: whether azithromycin administered to infants concurrently with the first and third doses of pentavalent vaccine (Penta-1 and Penta-3) reduces mortality from the time of the first dose through 6- 12 months of life. Pregnant participants are randomized to receive 2g azithromycin or placebo as a single oral dose twice during pregnancy and at delivery, for up to three total doses. Their infants are randomized to receive 20 mg/kg azithromycin or placebo as a single oral dose at their routine Penta-1 and Penta-3 vaccination visits (Figure 1). According to the national routine immunization schedule, these vaccination visits occur at approximately 6 and 14 weeks of age, respectively. Infants are followed through at least 6 months and up to 12 months of age. To increase statistical power for aim 1B, a separate cohort of 8,500 infants, the infant-only cohort, is recruited at the Penta-1 visit. Infants enrolled in the infant-only cohort are randomized, dosed, and followed in the same manner as those born to pregnant participants. Participant enrollment commenced in September 2020 and is expected to be complete by April 2023.

We hypothesize that the composite outcome of stillbirths and infant deaths will be lower in infants whose mothers were randomized to receive azithromycin in pregnancy compared to those randomized to receive placebo, and that mortality will be lower in infants who were randomized to receive azithromycin at first and third pentavalent vaccination visits compared to those randomized to receive placebo.

The trial has been registered at ClinicalTrials.gov (NCT03909737). The WHO trial registration data set can be found online in the supplemental appendix, Table 1.

For peer review only - http://bmjopen.bmj.com/site/about/guidelines.xhtml

Study Setting

SANTE is conducted by investigators at the Centre pour le Développement des Vaccins, Mali (CVD-Mali), an ongoing collaboration between the University of Maryland School of Medicine and the Malian Ministry of Health. For the past decade, much of northern and central Mali has been besieged by political instability and violence. The southwestern region of Sikasso, recently divided into the independent regions of Sikasso and Koutiala, had a reported infant mortality rate of 67 per 1000 live births in 2018.(15) It was selected as the study setting due to its high under-five mortality, moderate utilization of antenatal care and routine immunization coverage, and relative security (Supplemental Appendix, Table 2). Malaria is endemic in southwestern Mali, and standard of care for pregnant women is at least three doses of sulfadoxine-pyrimethamine (SP) intermittent preventive treatment (IPTp). Children aged 3-59 months receive a combination of monthly SP plus amodiaquine as seasonal malaria chemoprophylaxis (SMC) during peak malaria transmission season (July-October). In Sikasso, IPTp coverage with three or more doses is 26.8%, and 54% of age-eligible children receive one or more doses of SMC each year.(15) Within the Koutiala and Sikasso regions, the districts of Koutiala, Kignan, and Niena were selected for study operations. These districts represent areas with a high burden of pediatric illness and have populations of sufficient size to meet the study's enrollment requirement within the target timeframe.

Recruitment

Mali's decentralized health system operates at national, regional, district (*cercle*), health area (*aire de santé*), and community levels. Each district is served by a referral center (*Centre de Santé de Référence*, CSREF) and each *aire de santé* is served by one or more community health centers (*Centres de Santé Communautaire*, CSCOMs). SANTE study teams are embedded at all operational and accessible CSREFs and CSCOMs in the Koutiala, Kignan, and Niena districts. Many rural CSCOMs conduct regular community outreach operations (*stratégie avancée*), sending mobile units to remote health outposts to provide ANC and immunization services. Study participants are recruited at antenatal or immunization visits at participating CSREFs and CSCOMs, or from remote health posts as part of the *stratégie avancée*.

Inclusion and Exclusion Criteria

Pregnant individuals seeking ANC are eligible to be enrolled if they have reached at least 14 weeks gestation and are not in active labor. If ultrasound dating is unavailable, participants are deemed eligible if the fundus can be palpated at two or more finger breadths above the pubic symphysis, a proxy measurement for a gestational age of at least 14 weeks.(16) Infants in the infant-only cohort are eligible

For peer review only - http://bmjopen.bmj.com/site/about/guidelines.xhtml
For peer review only

to be enrolled if they are at least six weeks and fewer than 12 months of age and are presenting to a study facility to receive Penta-1 vaccine. Additional enrollment criteria are provided in Table 1.

Table 1. Inclusion and Exclusion Criteria

Inclusion Criteria	Exclusion Criteria
**Enrollment Criteria: Pregnant women and unborn infants enrolled in mother-infant cohort**	
• Participant is estimated to be ≥14 weeks gestation according to the study algorithm and is presenting at a study facility<br>• Participant understands and can comply with planned study procedures, as determined by the study staff<br>• Participant or legal authority has provided informed consent prior to initiation of any study procedures<br>• Participant intends to reside in the study area until her newborn infant is at least 6 months old	• Participant is in active labor, as defined by study standard operating procedures<br>• Participant has a known allergy to macrolide antibiotics<br>• Participant has a condition that, in the opinion of the investigator, might compromise his or her well-being, or affect the ability of the participant to comply with study procedures<br>• Participant is currently being treated with a macrolide antibiotic for a medical condition (this can be a temporary exclusion if the drug is later discontinued)
**Enrollment Criteria: Infants enrolled in the infant-only cohort**	
• Participant is presenting for Penta-1 vaccination at a study facility<br>• Participant is at least 6 weeks of age, and fewer than 12 months of age<br>• Participant's parent/guardian understands and can comply with the study procedures, as determined by the study staff<br>• Participant's parent or guardian has provided informed consent prior to initiation of any study procedures<br>• Participant's parent or guardian intends to reside in the study area until infant is at least 6 months old	• Participant has a known allergy to macrolide antibiotics<br>• Participant has a condition that, in the opinion of the investigator, might compromise his or her well-being, or affect the ability of the participant to comply with study procedures<br>• Participant is currently being treated with a macrolide antibiotic for a medical condition (this can be a temporary exclusion if the drug is later discontinued)

Randomization and masking

Treatment allocation is stratified by enrollment facility and randomized in permuted blocks of varying sizes. Participants are randomized 1:1 to one of the treatment assignments, with four possible assignments in the mother-infant cohort and two possible assignments in the infant-only cohort (**Figure**

For peer review only - http://bmjopen.bmj.com/site/about/guidelines.xhtml

1). Infants born to mothers enrolled in the mother-infant cohort are randomized when the mother is enrolled, i.e., before the infant is born.

Participants are randomized at enrollment using barcoded labels that are pre-printed with a sequential participant identification number and randomization assignment. The randomization assignment corresponds to a masked treatment bin, denoted by a letter of the alphabet, and pre-printed on every study medication bottle. Participants, field workers and study investigators are unaware of the link between the bin letters and the treatment assignments.

Intervention

Participants receive either azithromycin (Zithromax®) or placebo, both donated by the Pfizer Corporation. Both have the same appearance and color, but the placebo contains no active substance. Study medication is reconstituted at the time of dosing and administered under direct observation in oral suspension form.

Pregnant participants are dosed with 50mL of reconstituted liquid, equivalent to 2g of study medication. Infants are dosed 20 mg/kg up to a maximum of 1g, consistent with the recommended dosage for MDA.(9) Infant doses are automatically calculated by the electronic data collection form according to the infant's weight. Participants are observed for 30 minutes after receiving each dose. Participants who have an allergic reaction to the study medication are given medical attention as appropriate and are retained in the study but do not receive additional doses.

Dosing timepoints are aligned with routine healthcare visits. A minimum 72-hour window is required between doses. Pregnant participants receive up to three single doses over the course of the study, comprised of 1-2 antenatal doses and a dose at delivery (**Table 2**).

Table 2. Study Schedule

Timepoint	ANC visit ≥14 weeks gestation	Second ANC visit	Delivery	1 week after delivery	Penta-1 visit (approx. 6 weeks after delivery)	Penta-3 visit (approx. 14 weeks after delivery)	6 months after delivery	12 months after delivery[1]
**Mother-Infant Cohort participants**								
Eligibility assessment, and informed consent	M							
Randomization	M/I							
Administration of study medication	M	M	M		I	I		

For peer review only - http://bmjopen.bmj.com/site/about/guidelines.xhtml

Timepoint	ANC visit ≥14 weeks gestation	Second ANC visit	Delivery	1 week after delivery	Penta-1 visit (approx. 6 weeks after delivery)	Penta-3 visit (approx. 14 weeks after delivery)	6 months after delivery	12 months after delivery[1]
**Mother-Infant Cohort participants**								
Demographic Questionnaire	M							
Medical history	M	M	M		M/I	I		
Labor and Delivery Questionnaire			M/I					
Weight measurement			I		I	I		
Vital Status Assessment		M	M/I	I	M/I	I	I	I
**Infant-only Cohort Participants**								
Eligibility Assessment, informed consent, and randomization					I			
Administration of study medication					I	I		
Demographic Questionnaire					I			
Medical history					I	I		
Weight measurement					I	I		
Vital Status					I	I	I	I

ANC= antenatal care
Penta-1 = First pentavalent vaccination
Penta-3= Third pentavalent vaccination
M=Mother participant; I=Infant participant
[1]Infants who reach 12 months of age before the end of follow up have a final vital status assessment at 12 months. All other infants have a final vital status assessment at the end of the study follow up period when they are between 6 and 12 months of age.

Ideally, pregnant participants are dosed in the second and third trimesters, but to accommodate individual variations in ANC timing, the first dose is administered at the enrollment ANC visit and the second dose at the next routine ANC visit or at delivery, whichever occurs sooner. The delivery dose is given during labor when possible but may be administered within 24 hours after delivery regardless of whether the infant is stillborn or liveborn. Infants are dosed when they present to a study facility to receive their first and third doses of pentavalent vaccine.

Follow up and Primary Outcome measurements

The primary study outcomes are a composite of stillbirths and infant deaths for aim 1A, and infant deaths for aim 1B. Stillbirths are defined according to the WHO early stillbirth definition, i.e., the birth of a fetus with no signs of life at ≥22 weeks gestation, or the birth of a fetus with no signs of life weighing

For peer review only - http://bmjopen.bmj.com/site/about/guidelines.xhtml

at least 500g.(17) Birth outcomes are recorded for every enrolled participant at the delivery visit. Participants who miss the delivery visit are followed up at least three times to ascertain the birth outcome.

Infant deaths are defined as any death of a liveborn infant in the first year of life, with infant vital status assessed at multiple timepoints (**Table 2**). All enrolled infants are followed through at least six months of age. Study follow up ends when the last enrolled infant reaches six months of age. At that point, all infants between six and 12 months of age will have a final vital status assessment. Infants who reach 12 months of age before the end of the trial will have a final vital status assessment at 12 months. Participants with a six-month vital status that is unknown after at least three contact attempts by the study team are considered lost to follow-up.

Secondary Outcome measurements

Secondary outcomes include low birth weight (<2500g), gestational age at birth (Supplemental Appendix, Table 3), neonatal death (0-<28 days), and a composite outcome of maternal hospitalizations and mortality through six weeks postpartum. The cost effectiveness of the interventions will also be estimated as a secondary aim.

Safety monitoring and adverse events

Azithromycin is considered safe for use in pregnancy and is recommended for the treatment of urethritis and cervicitis caused by *Chlamydia trachomatis* and *Neisseria gonorrhoeae.* (18) Increased risks of malformations have been reported in some observational studies following first trimester exposure to azithromycin or to any macrolide antibiotic,(19-23) but not in others.(24-27) In a meta-analysis, azithromycin use (vs. alternative antibiotics) in the first trimester was weakly associated with gastrointestinal malformations.(28) In the second and third trimesters, macrolide exposure was associated with genital malformations, comprised mostly of hypospadias.(19) One case control study reported increased relative odds of miscarriage in the first 20 weeks of pregnancy among those prescribed azithromycin.(29) A limitation of these observational studies is the potential for confounding by indication. In SANTE, participants are enrolled after the first 14 weeks of pregnancy and study personnel are trained to identify and report major congenital malformations as serious adverse events (SAEs). Other events considered as SAEs include pregnancy losses before 22 weeks, maternal deaths, hospitalizations, and other significant or life-threatening illnesses. SAEs are reviewed by study physicians and assessed for relatedness to study medication.

For peer review only - http://bmjopen.bmj.com/site/about/guidelines.xhtml

In young children, the most common adverse reactions to azithromycin are gastrointestinal symptoms, reported in 1-4% of those treated.(9, 30) Cardiac toxicity is a potential concern, although the risk is not well-established in pediatric populations.(9) A randomized trial of 450 children aged six weeks to 59 months found no severe adverse events in azithromycin treated children, and only vomiting was reported more frequently in the azithromycin arm.(31) An association between treatment with erythromycin and infantile hypertrophic pyloric stenosis (IHPS) has been reported in the first two weeks of life, and to a lesser extent, in the third through fifth weeks of life.(32) The relationship between azithromycin and IHPS in infants less than six weeks of age is unclear.(33) In a recent RCT, one case of IHPS was reported among more than 10,000 azithromycin-treated infants.(34) The first dose of study medication in SANTE is administered at age 6 weeks or greater, although breastfed infants born to enrolled mothers could be exposed to low levels of azithromycin following the maternal delivery dose.(35) IHPS is monitored in SANTE as an adverse event of special interest and symptoms are solicited at all infant visits. An algorithm prompts physician review and assessment for relatedness, with any instances of probable or confirmed IHPS reported as SAEs (Supplemental Appendix, Figure 1).

To detect potential harmful associations between azithromycin and the SANTE study endpoints, an independent statistician analyzes stillbirths and infant deaths by treatment group at pre-specified intervals. The integrity of the trial is maintained by limiting these analyses to the harmful side of the sampling distribution of the hazard and risk ratio statistics. Results are reported to the data safety and monitoring board (DSMB).

Statistical Considerations

Sample size and power

The trial aims to enroll 33,600 mother-infant pairs plus 8,500 additional infants in the infant-only cohort. Power calculations for the primary aims were based on anticipated stillbirth and infant mortality rates in the Sikasso/Koutiala regions (**Table 3**). Analyzable endpoints are expected for at least 85% of enrolled participants.

Table 3. Detectable effect sizes and assumptions used for sample size calculations for primary aims

	**Aim 1A. Effect of intervention on composite outcome of stillbirths and infant deaths from birth to 12 months**
Total to enroll	33,600

For peer review only - http://bmjopen.bmj.com/site/about/guidelines.xhtml

Total expected analyzable	28,560	
	Minimum detectable relative effect	
	80% power	90% power
Baseline stillbirth and infant mortality rate 105/1000 births[1], 2012 DHS estimates	9.5%	10.9%
Baseline stillbirth and infant mortality rate 97/1000 births[2], 2018 DHS estimates	9.9%	11.4%
	**Aim 1B. Effect of intervention on infant deaths from first dose (approx. 6 weeks) to 12 months**	
	80% power	90% power
Baseline infant mortality rate 28/1000 live births, 2012 DHS estimates	18.6%	21.4%
With additional 7,650[3] from infant-only cohort	16.6%	19.1%
Baseline infant mortality rate 22/1000 live births, 2018 DHS estimates	20.9%	24.0%
With additional 7,650 from infant-only cohort	18.7%	21.5%

[1]33 stillbirths / 1000 births (36) and 74.8 infant deaths per 1000 live births (Sikasso data, Mali 2012 DHS report (37)), assuming that 6-11 month mortality data is available for 90% of infants and subtracting stillbirths from the live birth denominator.

[2]Infant mortality updated to 65.9 infant deaths per 1000 live births (Sikasso data, Mali 2018 DHS report (15)), other assumptions remain the same as in ([1]) above

[3]Assume 7,650 analyzable infants from 8,500 total enrolled in the rural infant-only cohort (10% LTFU)

Analysis of primary aims

The primary analysis for aim 1A will be according to the intention to treat (ITT) principle. For aim 1B, the primary analysis will be according to modified ITT, beginning from the first assigned dose. Sensitivity analyses of the two aims will be modified as-treated. In the 2x2 factorial design, the primary analyses will fit a Cox proportional hazards regression model with two indicator variables for the two interventions. The study is not powered to detect a statistical interaction effect between the two interventions, but if it is important (p<0.05 or ratio of hazard ratios >1. 5 or <0.67), results for all four study arms also will be reported. For primary aim 1A, the analysis timeline will be calculated from

For peer review only - http://bmjopen.bmj.com/site/about/guidelines.xhtml

For peer review only

delivery, with stillbirths assigned day 0.1. For primary aim 1B, the timeline will start at the receipt of the first infant dose. For a measure of overall public health impact on infant mortality, a supplementary analysis will be done with the timeline starting at the live birth.

Interim Analysis

There is one pre-specified formal interim analysis for efficacy planned for the maternal intervention, to take place when half of the expected number of stillbirths and infant deaths have occurred.

Cost Effectiveness Analysis

We will conduct a cost-effectiveness analysis of maternal, infant, and combination azithromycin delivery, reflecting the arms of the trial. The economic cost of program implementation will be estimated by summing the market price of azithromycin with costs of drug storage and personnel time for administration. Health outcomes will include maternal and infant mortality as measured for each arm, converted into disability-adjusted life-years (DALYs) based on life tables for Mali. We will calculate incremental cost-effectiveness ratios compared to the status quo and for progressively expensive strategies.

Ancillary Studies

Ancillary studies will be carried out in subsets of participants. Longitudinal sampling of mother-infant pairs will measure antimicrobial resistance of *Streptococcus pneumoniae* and *Escherichia coli* isolates and identify genomic markers of resistance. The impact of co-administration of azithromycin with rotavirus vaccine will be assessed. Several ancillary studies are aimed at elucidating the mechanisms by which azithromycin might prevent stillbirths and infant deaths, including investigations of the impact of azithromycin on maternal and infant anthropometry, the vaginal microbiome, the maternal and infant gut microbiomes, biomarkers of environmental enteric dysfunction, and malaria in pregnancy and infancy.

Data Collection and Management

All study data are collected electronically on tablets using the open-source Tangerine data collection platform. The data collection platform is developed and maintained by RTI International, who serves as the data coordinating center (DCC). Data are collected offline, and tablets are regularly synced to a cloud-based server for central aggregation and processing. Data are managed collaboratively by the DCC and study teams.

For peer review only - http://bmjopen.bmj.com/site/about/guidelines.xhtml

Community Engagement and Consent

Community engagement was initiated in the study planning phase and will continue throughout the trial period. Engagement takes place through a series of meetings including local health center personnel, religious and cultural leaders, and other community stakeholders at the regional and district levels. At each meeting, the investigators explain the trial procedures, the risks and benefits of participation, and other relevant information. A dynamic exchange ensues, and permission from the community leaders must be obtained to initiate and continue the trial.

Ethics and Dissemination

Written informed consent is obtained from the participant or their guardian. The study is approved by the ethics committees representing the University of Maryland, Baltimore, and the Faculté de Médecine et d'Odonto-Stomatologie in Mali. Results of the trial will be shared with the communities where participants were recruited, Mali's Ministry of Health, the WHO, and other stakeholders. A data sharing policy will be implemented. Results will be published in the peer-reviewed literature.

Study Oversight and Quality Assurance

A DSMB comprised of experts in pediatrics, obstetrics, biostatistics, and research ethics oversees patient safety and data collection for the trial. External monitoring visits are conducted quarterly. The study medication is analyzed on a routine basis by an independent laboratory.

Patient and Public Involvement

Local stakeholders in Mali will be involved in the research dissemination plans.

DISCUSSION

The SANTE trial is poised to address important knowledge gaps regarding the potential for azithromycin to improve pregnancy outcomes and infant survival in a high mortality setting. Unique attributes of SANTE compared to other RCTs of azithromycin in pregnancy include (1) a large sample size with statistical power to detect a reduction in stillbirths and infant mortality, (2) a combined antenatal and intrapartum intervention, and (3) the assessment of azithromycin alone rather than in combination with other antibiotics or as part of an iPTP regimen.

With antibacterial, antiprotozoal, antimalarial, immunomodulatory, and possibly antiviral properties, azithromycin delivered antenatally could prevent stillbirths and neonatal deaths by acting on causal or contributing factors including maternal infection, sterile or infection-related inflammation, and poor maternal nutritional status.(38-42) Support for these hypothesized mechanisms of action is provided by

For peer review only - http://bmjopen.bmj.com/site/about/guidelines.xhtml

randomized trials showing a reduction in low birthweight and preterm birth following antenatal azithromycin.(43-45) In The Gambia, a single 2g dose of intrapartum azithromycin reduced bacterial carriage and the incidence of non-severe infection in neonates.(14, 46) Whether this finding translates to reduced neonatal sepsis and death is being assessed in a follow up trial (NCT03199547). A separate multi-site trial will assess whether 2g intrapartum azithromycin prevents sepsis and death in neonates and their mothers (NCT03871491). We did not identify any published RCTs of azithromycin in pregnancy with stillbirth or infant death as the primary aim. Those that included a stillbirth outcome found no effect.(45, 47, 48) Three trials reported a reduction in neonatal (45, 49) or post-neonatal mortality (50) while others found no difference by mortality by treatment arm.(14, 43, 47, 51, 52)

Evidence of azithromycin's effect on child mortality comes from three large, randomized trials. The first reported a 49% reduction in all-cause mortality (95% CI 10, 71) among Ethiopian children 1-9 years residing in communities randomized to receive MDA-azithromycin compared to those in control communities.(10) The second, known as the MORDOR trial, found a 13.5% (95%CI 6.7, 19.8) reduction in mortality in children 1-59 months and a 24.9% (95% CI 10.6, 37.0) reduction in infants 1-5 months who resided in communities randomized to receive biannual MDA-azithromycin compared to placebo. (11) Although MORDOR was designed to detect a mortality effect across sites in three countries, a statistically significant benefit was only observed in Dosso, Niger, the site with the highest under-five mortality. To maximize the potential benefit of the infant intervention in SANTE, we situated the study in a region of Mali where the infant mortality rate is similar to that of the Dosso region.(53, 54) Targeted administration earlier in infancy, when the risk of death is greater, and the addition of a second dose within the first six months of life could potentially increase the survival benefit for infants in SANTE above that observed in MORDOR.

In contrast to the MDA-azithromycin trials, Chandramohan et al. reported no reduction in hospitalizations or mortality among children aged 3-59 months in Burkina Faso and Mali who were randomized at the household level to receive monthly SMC in combination with either azithromycin or placebo during the malaria transmission season.(12) One potential explanation for the difference in findings is that mortality reduction in the MORDOR sites, where routine SMC was not implemented, might have been due to azithromycin's antimalarial properties, and that there was no additional benefit of azithromycin beyond concurrent SMC in the Chandramohan trial. Children 3-59 months residing in the SANTE study area will be eligible to receive SMC during the malaria transmission season, but there are several reasons that SMC might be less influential in the SANTE trial. First, most infant deaths occur

For peer review only - http://bmjopen.bmj.com/site/about/guidelines.xhtml

before three months of age. Second, only a subset of infants enrolled in SANTE will reach three months of age during the malaria season and thus be eligible for SMC. Third, only 54% of caregivers in Sikasso reported that their age-eligible child received one or more SMC doses during the malaria season.(15) This suggests that SMC coverage among infants enrolled in SANTE will be considerably lower than in the Chandramohan trial, where it was administered to all participants as part of the study intervention.

Most recently, the NAITRE trial in Burkina Faso individually randomized infants at 8-27 days of age to receive a single oral dose of 20mg/kg azithromycin or placebo and found no reduction in mortality through six months of age.(34) Important differences between SANTE and NAITRE include different dosing schedules, higher baseline infant mortality in the SANTE study area, the inclusion of very remote populations in SANTE, and the inclusion of infants in the early neonatal period in SANTE.

The SANTE trial has two important limitations. The first is that ascertaining a reliable cause of death is not possible in the rural study setting. This limits inferences that can be made about differences in cause of death by treatment arm, although SANTE will conduct ancillary studies to understand possible mechanisms by which azithromycin might prevent mortality. Second, SANTE's individually randomized design will likely not achieve the interruption of pathogen transmission that might have occurred in MDA trials. One reason why a significant survival benefit for azithromycin has so far only been demonstrated in trials that randomized at the community level could be that the mechanism of protection requires high intervention coverage to interrupt pathogen transmission.(55)

Conclusion

SANTE is a large 2x2 factorial individually randomized placebo-controlled trial in a high mortality setting designed to generate evidence regarding the use of prophylactic azithromycin in pregnancy and early infancy to prevent stillbirths and infant deaths.

Acknowledgements

The authors would like to acknowledge the data safety and monitoring board and the SANTE study group and for their many contributions to this work:

Bill & Melinda Gates Foundation– Laura Lamberti

Data and Safety Monitoring Board – *Baylor College of Medicine* – Flor Muñoz-Rivas; *University of Washington School of Medicine* - Linda Eckert; *University of Mali, Bamako* – Allassane Dicko; *Faculté de*

For peer review only - http://bmjopen.bmj.com/site/about/guidelines.xhtml

Médicine Pharmacie et d'OdontoStomatologie – Saïbou Maiga; *University of California at San Francisco* – Travis Porco

SANTE Study Group - *University of Maryland, Baltimore* –Wilbur Chen, Fleesie Hubbard, Yuanyuan Liang, Jacques Ravel, Ousmane S. Sow, Sharon Tennant, Mark Travassos; *Centre pour le Développement des Vaccins, Bamako, Mali* –Mamadou Diallo, Jane Juma, Awa Traore - *RTI International* - Matthew Finholt-Daniel, Emily Hadley, Jennifer J. Hemingway-Foday, Chris Kelley, Alice Litavecz, Elizabeth McClure, Melissa Page, David Plotner, RJ (Steinert) Corwin, Kevin Wilson; *PATH* – Robert Choy; *Institute for Health Metrics and Evaluation, University of Washington, Seattle, WA, United States* -Michael Arndt.

Author contributions

Conceived and designed the study: KLK, MDT, SOS, LRM, AJD, ELD, FCH, RLG; Created tables/figures: AJD; Wrote first draft of manuscript: AJD; Read and critically revised the manuscript: All authors

Funding statement

This work was supported by the Bill & Melinda Gates Foundation grant number 1196125. The funders played no role in the design, writing, or decision to publish the study protocol.

Ethics Approval

Ethical approval was obtained for the SANTE study from the Institutional Review Boards at the University of Maryland, Baltimore (Protocol # HP- 00084242) and the Faculté de Médecine et d'Odonto-Stomatologie in Mali.

Competing Interests

Dr. Moulton serves on the Data Safety and Monitoring Board for Pfizer COVID-19 and respiratory syncytial virus vaccine trials.

Figure Legend

Figure 1. SANTE study design. Flow diagram to illustrate randomization and treatment groups for the 2x2 factorial mother-infant cohort and the infant-only cohort. "Penta 1" and "Penta 3" correspond to the first and third doses of pentavalent vaccine (i.e., diphtheria, tetanus, pertussis, hepatitis, and Hib-containing vaccine).

References

1. Estimation UI-aGfCM. Levels and Trends in Child Mortality - Report 2020. Geneva, Switzerland; 2020.
2. Global, regional, national, and selected subnational levels of stillbirths, neonatal, infant, and under-5 mortality, 1980-2015: a systematic analysis for the Global Burden of Disease Study 2015. Lancet (London, England). 2016;388(10053):1725-74.
3. UN Inter-agency group for Child Mortality Estimation. A Neglected Tragedy: The global burden of stillbirths. Geneva; 2020.

For peer review only - http://bmjopen.bmj.com/site/about/guidelines.xhtml

4. Reinebrant HE, Leisher SH, Coory M, Henry S, Wojcieszek AM, Gardener G, et al. Making stillbirths visible: a systematic review of globally reported causes of stillbirth. BJOG : an international journal of obstetrics and gynaecology. 2018;125(2):212-24.
5. Goldenberg RL, Harrison MS, McClure EM. Stillbirths: The Hidden Birth Asphyxia - US and Global Perspectives. Clinics in perinatology. 2016;43(3):439-53.
6. Goldenberg RL, Muhe L, Saleem S, Dhaded S, Goudar SS, Patterson J, et al. Criteria for assigning cause of death for stillbirths and neonatal deaths in research studies in low-middle income countries. The journal of maternal-fetal & neonatal medicine : the official journal of the European Association of Perinatal Medicine, the Federation of Asia and Oceania Perinatal Societies, the International Society of Perinatal Obstet. 2018:1-9.
7. McClure EM, Silver RM, Kim J, Ahmed I, Kallapur M, Ghanchi N, et al. Maternal infection and stillbirth: a review. The journal of maternal-fetal & neonatal medicine : the official journal of the European Association of Perinatal Medicine, the Federation of Asia and Oceania Perinatal Societies, the International Society of Perinatal Obstet. 2020:1-9.
8. Taylor HR, Burton MJ, Haddad D, West S, Wright H. Trachoma. Lancet (London, England). 2014;384(9960):2142-52.
9. Organization WH. WHO guideline on mass drug administration of azithromycin to children under five years of age to promote child survival 2020.
10. Porco TC, Gebre T, Ayele B, House J, Keenan J, Zhou Z, et al. Effect of mass distribution of azithromycin for trachoma control on overall mortality in Ethiopian children: a randomized trial. Jama. 2009;302(9):962-8.
11. Keenan JD, Bailey RL, West SK, Arzika AM, Hart J, Weaver J, et al. Azithromycin to Reduce Childhood Mortality in Sub-Saharan Africa. The New England journal of medicine. 2018;378(17):1583-92.
12. Chandramohan D, Dicko A, Zongo I, Sagara I, Cairns M, Kuepfer I, et al. Effect of Adding Azithromycin to Seasonal Malaria Chemoprevention. The New England journal of medicine. 2019;380(23):2197-206.
13. Hume-Nixon M, Quach A, Reyburn R, Nguyen C, Steer A, Russell F. A Systematic Review and meta-analysis of the effect of administration of azithromycin during pregnancy on perinatal and neonatal outcomes. EClinicalMedicine. 2021;40:101123.
14. Oluwalana C, Camara B, Bottomley C, Goodier S, Bojang A, Kampmann B, et al. Azithromycin in Labor Lowers Clinical Infections in Mothers and Newborns: A Double-Blind Trial. Pediatrics. 2017;139(2).
15. Institut National de la Statistique - INSTAT CdPedSSS-DSePdlFCS-D-PeI. Enquête Démographique et de Santé au Mali 2018. Bamako, Mali et Rockville, Maryland, USA : INSTAT, CPS/SS-DS-PF et ICF.; 2018.
16. Krelin Naidu KLF. Gestational Age Assessment [Updated 2021 Aug 2]. Treasure Island (FL): StatPearls Publishing; Jan 2022.
17. Organization WH. Making every baby count: audit and review of stillbirths and neonatal deaths at facility. Geneva; 2016.
18. Prevention CfDCa. Sexually Transmitted Infections Treatment Guidelines, 2021. 2021. Contract No.: 4.
19. Fan H, Gilbert R, O'Callaghan F, Li L. Associations between macrolide antibiotics prescribing during pregnancy and adverse child outcomes in the UK: population based cohort study. BMJ (Clinical research ed). 2020;368:m331.
20. Bojang A, Baines SL, Camara B, Guerillot R, Donovan L, Marqués RS, et al. Impact of Intrapartum Oral Azithromycin on the Acquired Macrolide Resistome of Infants' Nasopharynx: A Randomized Controlled Trial. Clinical infectious diseases : an official publication of the Infectious Diseases Society of America. 2020;71(12):3222-5.

For peer review only - http://bmjopen.bmj.com/site/about/guidelines.xhtml

21. Muanda FT, Sheehy O, Bérard A. Use of antibiotics during pregnancy and the risk of major congenital malformations: a population based cohort study. Br J Clin Pharmacol. 2017;83(11):2557-71.
22. Leke AZ, Dolk H, Loane M, Casson K, Nelen V, Barišić I, et al. Macrolide and lincosamide antibiotic exposure in the first trimester of pregnancy and risk of congenital anomaly: A European case-control study. Reprod Toxicol. 2021;100:101-8.
23. Mallah N, Tohidinik HR, Etminan M, Figueiras A, Takkouche B. Prenatal Exposure to Macrolides and Risk of Congenital Malformations: A Meta-Analysis. Drug Saf. 2020;43(3):211-21.
24. Andersson NW, Olsen RH, Andersen JT. Association between use of macrolides in pregnancy and risk of major birth defects: nationwide, register based cohort study. BMJ (Clinical research ed). 2021;372:n107.
25. Cooper WO, Hernandez-Diaz S, Arbogast PG, Dudley JA, Dyer SM, Gideon PS, et al. Antibiotics potentially used in response to bioterrorism and the risk of major congenital malformations. Paediatric and perinatal epidemiology. 2009;23(1):18-28.
26. Bérard A, Sheehy O, Zhao JP, Nordeng H. Use of macrolides during pregnancy and the risk of birth defects: a population-based study. Pharmacoepidemiol Drug Saf. 2015;24(12):1241-8.
27. Damkier P, Brønniche LMS, Korch-Frandsen JFB, Broe A. In utero exposure to antibiotics and risk of congenital malformations: a population-based study. American journal of obstetrics and gynecology. 2019;221(6):648.e1-.e15.
28. Fan H, Li L, Wijlaars L, Gilbert RE. Associations between use of macrolide antibiotics during pregnancy and adverse child outcomes: A systematic review and meta-analysis. PloS one. 2019;14(2):e0212212.
29. Muanda FT, Sheehy O, Bérard A. Use of antibiotics during pregnancy and risk of spontaneous abortion. Cmaj. 2017;189(17):E625-e33.
30. Zeng L, Xu P, Choonara I, Bo Z, Pan X, Li W, et al. Safety of azithromycin in pediatrics: a systematic review and meta-analysis. Eur J Clin Pharmacol. 2020;76(12):1709-21.
31. Sié A, Dah C, Bountogo M, Ouattara M, Nebie E, Coulibaly B, et al. Adverse Events and Clinic Visits following a Single Dose of Oral Azithromycin among Preschool Children: A Randomized Placebo-Controlled Trial. The American journal of tropical medicine and hygiene. 2020;104(3):1137-41.
32. Eberly MD, Eide MB, Thompson JL, Nylund CM. Azithromycin in early infancy and pyloric stenosis. Pediatrics. 2015;135(3):483-8.
33. Smith C, Egunsola O, Choonara I, Kotecha S, Jacqz-Aigrain E, Sammons H. Use and safety of azithromycin in neonates: a systematic review. BMJ open. 2015;5(12):e008194.
34. Oldenburg CE, Sié A, Bountogo M, Zakane A, Compaoré G, Ouedraogo T, et al. Neonatal Azithromycin Administration for Prevention of Infant Mortality. NEJM Evidence. 2022;1(4):EVIDoa2100054.
35. Azithromycin. Drugs and Lactation Database (LactMed). Bethesda (MD): National Library of Medicine (US); 2006.
36. Blencowe H, Cousens S, Jassir FB, Say L, Chou D, Mathers C, et al. National, regional, and worldwide estimates of stillbirth rates in 2015, with trends from 2000: a systematic analysis. The Lancet Global health. 2016;4(2):e98-e108.
37. DHS. Mali DHS, 2012-2013 Final Report. Rockville, MD: The DHS Program, USAID; 2012-2013.
38. Khoshnood S, Shirani M, Dalir A, Moradi M, Haddadi MH, Sadeghifard N, et al. Antiviral effects of azithromycin: A narrative review. Biomed Pharmacother. 2022;147:112682.
39. Kricker JA, Page CP, Gardarsson FR, Baldursson O, Gudjonsson T, Parnham MJ. Nonantimicrobial Actions of Macrolides: Overview and Perspectives for Future Development. Pharmacol Rev. 2021;73(4):233-62.
40. McClure EM, Goldenberg RL. Infection and stillbirth. Seminars in fetal & neonatal medicine. 2009;14(4):182-9.

For peer review only - http://bmjopen.bmj.com/site/about/guidelines.xhtml

41. Baker BC, Heazell AEP, Sibley C, Wright R, Bischof H, Beards F, et al. Hypoxia and oxidative stress induce sterile placental inflammation in vitro. Scientific reports. 2021;11(1):7281.
42. McClure EM, Saleem S, Pasha O, Goldenberg RL. Stillbirth in developing countries: a review of causes, risk factors and prevention strategies. The journal of maternal-fetal & neonatal medicine : the official journal of the European Association of Perinatal Medicine, the Federation of Asia and Oceania Perinatal Societies, the International Society of Perinatal Obstet. 2009;22(3):183-90.
43. Unger HW, Ome-Kaius M, Wangnapi RA, Umbers AJ, Hanieh S, Suen CS, et al. Sulphadoxine-pyrimethamine plus azithromycin for the prevention of low birthweight in Papua New Guinea: a randomised controlled trial. BMC medicine. 2015;13:9.
44. Luntamo M, Kulmala T, Mbewe B, Cheung YB, Maleta K, Ashorn P. Effect of repeated treatment of pregnant women with sulfadoxine-pyrimethamine and azithromycin on preterm delivery in Malawi: a randomized controlled trial. The American journal of tropical medicine and hygiene. 2010;83(6):1212-20.
45. Gray RH, Wabwire-Mangen F, Kigozi G, Sewankambo NK, Serwadda D, Moulton LH, et al. Randomized trial of presumptive sexually transmitted disease therapy during pregnancy in Rakai, Uganda. American journal of obstetrics and gynecology. 2001;185(5):1209-17.
46. Roca A, Oluwalana C, Bojang A, Camara B, Kampmann B, Bailey R, et al. Oral azithromycin given during labour decreases bacterial carriage in the mothers and their offspring: a double-blind randomized trial. Clinical microbiology and infection : the official publication of the European Society of Clinical Microbiology and Infectious Diseases. 2016;22(6):565.e1-9.
47. Kimani J, Phiri K, Kamiza S, Duparc S, Ayoub A, Rojo R, et al. Efficacy and Safety of Azithromycin-Chloroquine versus Sulfadoxine-Pyrimethamine for Intermittent Preventive Treatment of Plasmodium falciparum Malaria Infection in Pregnant Women in Africa: An Open-Label, Randomized Trial. PloS one. 2016;11(6):e0157045.
48. Moore BR, Benjamin JM, Tobe R, Ome-Kaius M, Yadi G, Kasian B, et al. A Randomized Open-Label Evaluation of the Antimalarial Prophylactic Efficacy of Azithromycin-Piperaquine versus Sulfadoxine-Pyrimethamine in Pregnant Papua New Guinean Women. Antimicrobial agents and chemotherapy. 2019;63(10):e00302-19.
49. Hendrixson DT, Smith K, Lasowski P, Callaghan-Gillespie M, Weber J, Papathakis P, et al. A novel intervention combining supplementary food and infection control measures to improve birth outcomes in undernourished pregnant women in Sierra Leone: A randomized, controlled clinical effectiveness trial. PLoS medicine. 2021;18(9):e1003618.
50. Hallamaa L, Cheung YB, Maleta K, Luntamo M, Ashorn U, Gladstone M, et al. Child Health Outcomes After Presumptive Infection Treatment in Pregnant Women: A Randomized Trial. Pediatrics. 2018;141(3).
51. Tita AT, Szychowski JM, Boggess K, Saade G, Longo S, Clark E, et al. Adjunctive Azithromycin Prophylaxis for Cesarean Delivery. The New England journal of medicine. 2016;375(13):1231-41.
52. Kalilani L, Mofolo I, Chaponda M, Rogerson SJ, Alker AP, Kwiek JJ, et al. A randomized controlled pilot trial of azithromycin or artesunate added to sulfadoxine-pyrimethamine as treatment for malaria in pregnant women. PloS one. 2007;2(11):e1166.
53. International INdlS-INaI. Enquête Démographique et de Santé et à Indicateurs Multiples du Niger 2012. Calverton, Maryland, USA; 2013.
54. DHS. Mali DHS, Final Report 2012-2013. Rockville, MD: The DHS Program, USAID; 2012-2013.
55. O'Brien KS, Oldenburg CE. MDA and trial designs to evaluate the impact of azithromycin on child mortality. The Lancet Global health. 2022;10(2):e183.

For peer review only - http://bmjopen.bmj.com/site/about/guidelines.xhtml

SANTE Protocol– FIGURE – August 8, 2022

Figure 1.

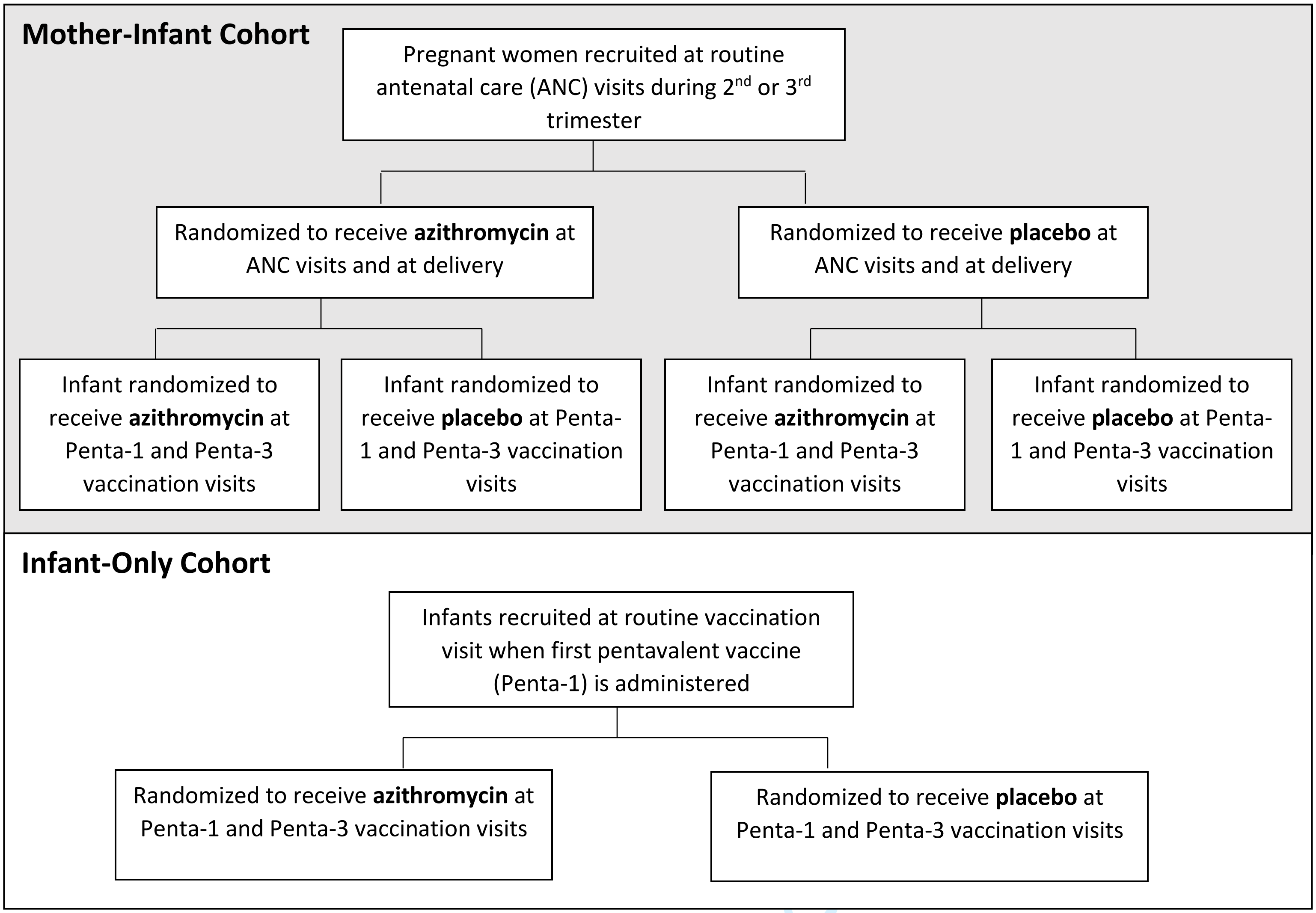

For peer review only - http://bmjopen.bmj.com/site/about/guidelines.xhtml

SANTE Protocol Manuscript – SUPPLEMENTAL APPENDIX – August 8, 2022

Supplemental Online Appendix.

Supplemental Table 1. World Health Organization Trial Registration Data Set

**Primary Registry and Trial Identifying Number**	ClinicalTrials.gov; NCT03909737
**Date of Registration in Primary Registry**	April 10, 2019
**Secondary Identifying Numbers**	
**Source(s) of Monetary or Material Support**	Bill & Melinda Gates Foundation
**Primary Sponsor**	University of Maryland, Baltimore
**Secondary Sponsor(s)**	n/a
**Contact for Public Queries**	Amanda Driscoll PhD<br>adriscoll@som.umaryland.edu
**Contact for Scientific Queries**	Karen Kotloff MD<br>Karen Kotloff kkotloff@som.umaryland.edu
**Public Title**	The SANTE trial
**Scientific Title**	Sauver avec l'Azithromycine en Traitant les Femmes Enceintes et les Enfants / Save with Azithromycin by Treating Pregnant Women and Children (SANTE)
**Countries of Recruitment**	Mali
**Health conditions or problems studied**	The burden of stillbirths and infant mortality remains high in low-income settings. Prophylactic azithromycin delivered in pregnancy and in infancy has shown promise as a potential intervention to improve birth outcomes and infant survival.
**Interventions**	Pregnant women: a single 2g dose of oral azithromycin (or placebo) delivered in the second and third trimesters of pregnancy, and at delivery<br>Infants: a single 20mg/kg dose of oral azithromycin (or placebo) delivered concurrently with the first and third doses of diphtheria, tetanus, pertussis containing vaccine.
**Key Inclusion and Inclusion Criteria**	At enrollment, pregnant women must be ≥14 weeks gestation and must not be in active labor. Infant participants must be ≥6 weeks of age at enrollment and/or the time of the first dose. Participants must plan to reside in the study area for at least 6 months after the infant is born. Participants who are being treated with azithromycin or another macrolide antibiotic for a medical condition are excluded from enrollment.
**Study Type**	Individually randomized 2x2 factorial placebo-controlled trial
**Date of First Enrollment**	September 2020
**Sample size**	33,600 mother-infant pairs, plus an additional 8,500 infants

For peer review only - http://bmjopen.bmj.com/site/about/guidelines.xhtml

**Recruitment Status**	Recruiting
**Primary Outcome(s)**	Primary outcome 1A: a composite of stillbirths and infant mortality within the first 6-12 months of life<br>Primary outcome 1B: infant mortality between the time of first does through 6-12 months of life
**Key Secondary Outcomes**	Low birth weight (<2500g), neonatal death (0-<28 days), and a composite outcome of maternal hospitalizations and mortality through six weeks postpartum. The cost effectiveness of the interventions will also be estimated as a secondary aim.
**Ethics Review**	This trial was approved by the Institutional Review Board at the University of Maryland School of Medicine (Protocol #HP-00084242) and the Faculté de Médecine et d'Odonto-Stomatologie in Mali
**Completion Date**	n/a
**Summary Results**	n/a
**IPD Sharing Statement**	Access to trial IPD may be provided to qualified researchers upon request and following review and approval of a research proposal and execution of a Data Sharing Agreement (DSA).

For peer review only

Supplemental Table 2. Characteristics of the Study Settings

	Sikasso Region (SANTE study area)	Comparator Indicators		
		Mali, national level indicators	African Region	Global
Under-five mortality rate per 1000 live births	111[1]	101[1]	76[2]	38[2]
Infant mortality rate per 1000 live births	67[1]	54[1]	52[2]	28[2]
Neonatal mortality rate per 1000 live births	42[1]	33[1]	27[2]	17[2]

SANTE Protocol Manuscript – SUPPLEMENTAL APPENDIX – August 8, 2022

Perinatal deaths per 1000 births (stillbirths plus early neonatal deaths w/in 7 days)[2]	45[3]	38[3]		
Stillbirths per 1000 births	7[3]	12[3] / 19.7[4]	21.7[4]	13.9[4]
Proportion of pregnancies where antenatal care was received by a trained provider	76.1%[5]	79.5%[5]		
Proportion of pregnant women that received 3+ doses of IPTp	26.8%[6]	28.3%[6]	35%[7]	
Proportion of births that were facility-based	71.0%[5]	66.8%[5]		
Proportion of births attended by a skilled provider	70.8%[5]	67.3%[5]		
Proportion of children 12-35 months of age who received DTP-1	85.7%[8]	82.1%[8]		
Proportion of children 12-35 months of age who received DTP-3	69.4%[8]	70.7%[8]		

[1]From Mali 2018 DHS Report, Tables 8.1 and 8.2

[2] 2019 data, from Levels & Trends in Child Mortality. Report 2020. Estimates developed by the UN Inter-agency Group for Child Mortality Estimation.

[3]From Mali 2018 DHS Report, Table 8.4. Stillbirths calculated as the difference between perinatal and early neonatal deaths, and defined as fetal deaths occurring at 7 months or more of pregnancy

[4] 2019 estimates from: Global, regional, and national estimates and trends in stillbirths from 2000 to 2019: a systematic assessment. 2021. Lancet 398:772-785. Stillbirth defined as fetal death at 28 weeks gestation or more.

[5]From Mali 2018 DHS Report, Tables 9.1, 9.5 and 9.6 – Trained providers defined as doctors, nurses, and midwives.

[6]IPTp= Intermittent preventive treatment for malaria during pregnancy. From Mali 2018 DHS Report, Table 12.9

[7]2019 estimate from World Malaria Report, 2021. World Health Organization. https://www.who.int/teams/global-malaria-programme/reports/world-malaria-report-2021

[8] DTP=Diphtheria, tetanus and pertussis containing vaccine. From Mali 2018 DHS Report, Table 10.3

[9]

For peer review only

Supplemental Table 3. Gestational Age Algorithm*

**For participants with only fundal health (FH) measurement available:**	
If FH measurement ≥16 cm or ≥35 cm	Assume cm FH = weeks gestational age
If FH measurement <16 cm	Assume GA= 14-15 weeks
If FH measurement >35 cm	Assume GA>35 weeks
**For participants with date of last menstrual period (LMP date) available:**	
If no FH or ultrasound dating (US) available	Use LMP date
If no US available and difference between LMP date and FH is +/- 14 days	Use LMP date
If no US available and difference between LMP date and FH is > 14 days	Use FH
If US is available:	
Earliest US at <9 weeks	
Within +/- 5 days of LMP date	Use LMP date
> 5 days different from LMP date	Use US
Earliest US at 9 to <16 weeks	
Within +/- 7 days of LMP date	Use LMP date
> 7 days different from LMP date	Use US
Earliest US at 16 to <22 weeks	
Within +/- 10 days of LMP date	Use LMP date
> 10 days different from LMP date	Use US
Earliest US at 22 to <28 weeks	
Within +/- 14 days of LMP date	Use LMP date
> 14 days different from LMP date	Use US
Earliest US at ≥28 weeks	
Within +/- 21 days of LMP date	Use LMP date
> 21 days different from LMP date	Use US
**For participants without FH, LMP date, or US**	Estimated GA is missing

*Adapted with permission from the A-PLUS study

For peer review only

For peer review only - http://bmjopen.bmj.com/site/about/guidelines.xhtml

SANTE Protocol Manuscript – SUPPLEMENTAL APPENDIX – August 8, 2022

Supplemental Figure 1. Infantile Hypertrophic Pyloric Stenosis Reporting Algorithm

Asked at each infant visit: Has the infant had forceful vomiting after feeding for 2 or more days, with vomit the color of breastmilk?

If yes, complete Adverse Event of Special Interest Form, shown below

**1.** Date of notification:	___ ___/___ ___ ___/___ ___ ___ ___ (dd/MMM/yyyy)
**2.** Date symptoms began	___ ___ ___ ___ Year (*enter 9999 if unknown*)<br>___ ___ ___ Month (*enter XXX if unknown*)<br>___ ___ Day (*enter 99 if unknown*)
**3.** Are the symptoms ongoing?	☐ YES (*skip to 4*)<br>☐ NO<br>☐ Unknown (*skip to 4*)
**3a**. (If NO to Q3) End date of symptoms	___ ___ ___ ___ Year (*enter 9999 if unknown*)<br>___ ___ ___ Month (*enter XXX if unknown*)<br>___ ___ Day (*enter 99 if unknown*)
**4.** Date of the last dose of study drug:*	___ ___/___ ___ ___/___ ___ ___ ___<br>(dd/MMM/yyyy)
**Event information**	
**5.** Age of the infant participant*	___ ___ weeks
**6.** Sex of the infant participant*	☐ Male ☐ Female ☐ Indeterminate
**7.** How many consecutive days did the vomiting last?	_______ Days
**8.** Does the infant participant seem to be hungry most of the day? (*select one option*)	☐ YES ☐ NO ☐ Unknown
**9.** Has the infant participant had diarrhea?	☐ YES ☐ NO ☐ Unknown
**10.** Did the parent/caregiver seek medical care for this vomiting episode? (*If NO, refer for a medical evaluation if the symptoms are ongoing*)	☐ YES ☐ NO ☐ Unknown
**10a**. (If YES to Q10) What was the diagnosis? (*select one option*)	☐ Pyloric stenosis<br>☐ Gastroenteritis<br>☐ Other, specify: __________<br>☐ Unknown
**10b**. (If YES to Q10) How was the diagnosis made? (*choose all options that apply*)	☐ Clinical exam (feeling a mass)<br>☐ Ultrasound<br>☐ Surgery<br>☐ Other, specify ______________<br>☐ Unknown
**10c.** (If YES to Q10) What care was provided? (*choose all options that apply*)	☐ Medicine<br>☐ Surgery<br>☐ None<br>☐ Unknown
**11.** Pyloric stenosis diagnosis*	☐ Possible (Forceful vomiting of 2 days or more color of breastmilk)<br>☐ Probable (Possible, with palpitation of a mass) (*Complete SAE form*)<br>☐ Definite (Sonogram, ultrasound, or surgical diagnosis) (*Complete SAE form*)

For peer review only - http://bmjopen.bmj.com/site/about/guidelines.xhtml

Reporting checklist for protocol of a clinical trial.

Based on the SPIRIT guidelines.

Instructions to authors

Complete this checklist by entering the page numbers from your manuscript where readers will find each of the items listed below.

Your article may not currently address all the items on the checklist. Please modify your text to include the missing information. If you are certain that an item does not apply, please write "n/a" and provide a short explanation.

Upload your completed checklist as an extra file when you submit to a journal.

In your methods section, say that you used the SPIRITreporting guidelines, and cite them as:

Chan A-W, Tetzlaff JM, Gøtzsche PC, Altman DG, Mann H, Berlin J, Dickersin K, Hróbjartsson A, Schulz KF, Parulekar WR, Krleža-Jerić K, Laupacis A, Moher D. SPIRIT 2013 Explanation and Elaboration: Guidance for protocols of clinical trials. BMJ. 2013;346:e7586

		Reporting Item	Page Number
**Administrative information**			
Title	#1	Descriptive title identifying the study design, population, interventions, and, if applicable, trial acronym	1

For peer review only

Trial registration	#2a	Trial identifier and registry name. If not yet registered, name of intended registry	1
Trial registration: data set	#2b	All items from the World Health Organization Trial Registration Data Set	S1-2
Protocol version	#3	Date and version identifier	n/a
Funding	#4	Sources and types of financial, material, and other support	13
Roles and responsibilities: contributorship	#5a	Names, affiliations, and roles of protocol contributors	1,13
Roles and responsibilities: sponsor contact information	#5b	Name and contact information for the trial sponsor	n/a
Roles and responsibilities: sponsor and funder	#5c	Role of study sponsor and funders, if any, in study design; collection, management, analysis, and interpretation of data; writing of the report; and the decision to submit the report for publication, including whether they will have ultimate authority over any of these activities	13
Roles and responsibilities: committees	#5d	Composition, roles, and responsibilities of the coordinating centre, steering committee, endpoint adjudication committee, data management team, and	11

For peer review only

		other individuals or groups overseeing the trial, if applicable (see Item 21a for data monitoring committee)	
**Introduction**			
Background and rationale	#6a	Description of research question and justification for undertaking the trial, including summary of relevant studies (published and unpublished) examining benefits and harms for each intervention	3, 8
Background and rationale: choice of comparators	#6b	Explanation for choice of comparators	3
Objectives	#7	Specific objectives or hypotheses	3
Trial design	#8	Description of trial design including type of trial (eg, parallel group, crossover, factorial, single group), allocation ratio, and framework (eg, superiority, equivalence, non-inferiority, exploratory)	3-4
**Methods: Participants, interventions, and outcomes**			
Study setting	#9	Description of study settings (eg, community clinic, academic hospital) and list of countries where data will be collected. Reference to where list of study sites can be obtained	4

For peer review only

Eligibility criteria	#10	Inclusion and exclusion criteria for participants. If applicable, eligibility criteria for study centres and individuals who will perform the interventions (eg, surgeons, psychotherapists)	4-6
Interventions: description	#11a	Interventions for each group with sufficient detail to allow replication, including how and when they will be administered	6
Interventions: modifications	#11b	Criteria for discontinuing or modifying allocated interventions for a given trial participant (eg, drug dose change in response to harms, participant request, or improving / worsening disease)	6
Interventions: adherance	#11c	Strategies to improve adherence to intervention protocols, and any procedures for monitoring adherence (eg, drug tablet return; laboratory tests)	7
Interventions: concomitant care	#11d	Relevant concomitant care and interventions that are permitted or prohibited during the trial	n/a
Outcomes	#12	Primary, secondary, and other outcomes, including the specific measurement variable (eg, systolic blood pressure), analysis metric (eg, change from baseline, final value, time to event), method of aggregation (eg, median, proportion), and time point for each outcome. Explanation of the clinical relevance of chosen efficacy and harm outcomes is strongly recommended	7-8

For peer review only

Participant timeline	#13	Time schedule of enrolment, interventions (including any run-ins and washouts), assessments, and visits for participants. A schematic diagram is highly recommended (see Figure)	6-7
Sample size	#14	Estimated number of participants needed to achieve study objectives and how it was determined, including clinical and statistical assumptions supporting any sample size calculations	8-9
Recruitment	#15	Strategies for achieving adequate participant enrolment to reach target sample size	4
**Methods: Assignment of interventions (for controlled trials)**			
Allocation: sequence generation	#16a	Method of generating the allocation sequence (eg, computer-generated random numbers), and list of any factors for stratification. To reduce predictability of a random sequence, details of any planned restriction (eg, blocking) should be provided in a separate document that is unavailable to those who enrol participants or assign interventions	5-6
Allocation concealment mechanism	#16b	Mechanism of implementing the allocation sequence (eg, central telephone; sequentially numbered, opaque,	5-6

For peer review only

		sealed envelopes), describing any steps to conceal the sequence until interventions are assigned	
Allocation: implementation	#16c	Who will generate the allocation sequence, who will enrol participants, and who will assign participants to interventions	5-6
Blinding (masking)	#17a	Who will be blinded after assignment to interventions (eg, trial participants, care providers, outcome assessors, data analysts), and how	5-6
Blinding (masking): emergency unblinding	#17b	If blinded, circumstances under which unblinding is permissible, and procedure for revealing a participant's allocated intervention during the trial	n/a
**Methods: Data collection, management, and analysis**			
Data collection plan	#18a	Plans for assessment and collection of outcome, baseline, and other trial data, including any related processes to promote data quality (eg, duplicate measurements, training of assessors) and a description of study instruments (eg, questionnaires, laboratory tests) along with their reliability and validity, if known. Reference to where data collection forms can be found, if not in the protocol	10

For peer review only

Data collection plan: retention	#18b	Plans to promote participant retention and complete follow-up, including list of any outcome data to be collected for participants who discontinue or deviate from intervention protocols	7
Data management	#19	Plans for data entry, coding, security, and storage, including any related processes to promote data quality (eg, double data entry; range checks for data values). Reference to where details of data management procedures can be found, if not in the protocol	10
Statistics: outcomes	#20a	Statistical methods for analysing primary and secondary outcomes. Reference to where other details of the statistical analysis plan can be found, if not in the protocol	8-10
Statistics: additional analyses	#20b	Methods for any additional analyses (eg, subgroup and adjusted analyses)	8-10
Statistics: analysis population and missing data	#20c	Definition of analysis population relating to protocol non-adherence (eg, as randomised analysis), and any statistical methods to handle missing data (eg, multiple imputation)	8-10

Methods: Monitoring

Data monitoring: formal committee	#21a	Composition of data monitoring committee (DMC); summary of its role and reporting structure; statement of whether it is independent from the sponsor and competing interests; and reference to where further	11

For peer review only

		details about its charter can be found, if not in the protocol. Alternatively, an explanation of why a DMC is not needed	
Data monitoring: interim analysis	#21b	Description of any interim analyses and stopping guidelines, including who will have access to these interim results and make the final decision to terminate the trial	10
Harms	#22	Plans for collecting, assessing, reporting, and managing solicited and spontaneously reported adverse events and other unintended effects of trial interventions or trial conduct	8
Auditing	#23	Frequency and procedures for auditing trial conduct, if any, and whether the process will be independent from investigators and the sponsor	11
**Ethics and dissemination**			
Research ethics approval	#24	Plans for seeking research ethics committee / institutional review board (REC / IRB) approval	11
Protocol amendments	#25	Plans for communicating important protocol modifications (eg, changes to eligibility criteria, outcomes, analyses) to relevant parties (eg, investigators, REC / IRBs, trial participants, trial registries, journals, regulators)	n/a

For peer review only

Consent or assent	#26a	Who will obtain informed consent or assent from potential trial participants or authorised surrogates, and how (see Item 32)	11
Consent or assent: ancillary studies	#26b	Additional consent provisions for collection and use of participant data and biological specimens in ancillary studies, if applicable	n/a
Confidentiality	#27	How personal information about potential and enrolled participants will be collected, shared, and maintained in order to protect confidentiality before, during, and after the trial	n/a
Declaration of interests	#28	Financial and other competing interests for principal investigators for the overall trial and each study site	13
Data access	#29	Statement of who will have access to the final trial dataset, and disclosure of contractual agreements that limit such access for investigators	S2
Ancillary and post trial care	#30	Provisions, if any, for ancillary and post-trial care, and for compensation to those who suffer harm from trial participation	n/a
Dissemination policy: trial results	#31a	Plans for investigators and sponsor to communicate trial results to participants, healthcare professionals, the public, and other relevant groups (eg, via publication, reporting in results databases, or other data sharing arrangements), including any publication restrictions	11

For peer review only

Dissemination policy: authorship	#31b	Authorship eligibility guidelines and any intended use of professional writers	n/a
Dissemination policy: reproducible research	#31c	Plans, if any, for granting public access to the full protocol, participant-level dataset, and statistical code	S2

Appendices

Informed consent materials	#32	Model consent form and other related documentation given to participants and authorised surrogates	n/a
Biological specimens	#33	Plans for collection, laboratory evaluation, and storage of biological specimens for genetic or molecular analysis in the current trial and for future use in ancillary studies, if applicable	n/a

The SPIRIT Explanation and Elaboration paper is distributed under the terms of the Creative Commons Attribution License CC-BY-NC. This checklist was completed on 17. August 2022 using https://www.goodreports.org/, a tool made by the EQUATOR Network in collaboration with Penelope.ai

For peer review only

BMJ Open

Antenatal, intrapartum, and infant azithromycin to prevent stillbirths and infant deaths: study protocol for SANTE, a 2x2 factorial randomized controlled trial in Mali

Journal:	*BMJ Open*
Manuscript ID	bmjopen-2022-067581.R1
Article Type:	Protocol
Date Submitted by the Author:	22-Dec-2022
Complete List of Authors:	Driscoll, Amanda; University of Maryland School of Medicine, Center for Vaccine Development and Global Health<br>Haidara, Fadima; Center for Vaccine Development–Mali<br>Tapia, Milagritos; University of Maryland School of Medicine, Center for Vaccine Development and Global Health<br>Deichsel, Emily L.; University of Maryland School of Medicine, Center for Vaccine Development and Global Health<br>Samake, Ousmane S.; Centre pour le Développement des Vaccins<br>Bocoum, Tiecoura; Centre pour le Développement des Vaccins<br>Bailey, Jason A.; University of Maryland School of Medicine, Center for Vaccine Development and Global Health<br>Fitzpatrick, Meagan ; University of Maryland School of Medicine, Center for Vaccine Development and Global Health<br>Goldenberg, Robert ; Columbia University School of Medicine, Obstetrics and Gynecology<br>Kodio, Mamoudou; Centre pour le Développement des Vaccins<br>Moulton, Lawrence H.; Johns Hopkins Bloomberg School of Public Health<br>Nasrin, Dilruba; University of Maryland School of Medicine, Department of Medicine<br>Onwuchekwa, Uma; Centre pour le Développement des Vaccins<br>Shaffer, Allison M.; University of Maryland School of Medicine, Center for Vaccine Development and Global Health<br>Sow, Samba O.; Center for Vaccine Development; Centre pour le Développement des Vaccins<br>Kotloff , Karen ; University of Maryland School of Medicine, Center for Vaccine Development and Global Health
**Primary Subject Heading**:	Global health
Secondary Subject Heading:	Infectious diseases
Keywords:	INFECTIOUS DISEASES, PERINATOLOGY, PAEDIATRICS, EPIDEMIOLOGY

SCHOLARONE™
Manuscripts

Antenatal, intrapartum, and infant azithromycin to prevent stillbirths and infant deaths: study protocol for SANTE, a 2x2 factorial randomized controlled trial in Mali

Amanda J. Driscoll[1]*, Fadima C. Haidara[2]*, Milagritos D. Tapia[1,5], Emily L. Deichsel[1], Ousmane S. Samake[2], Tiecoura Bocoum[2], Jason A. Bailey[1], Meagan C. Fitzpatrick[5], Robert L. Goldenberg[4], Mamoudou Kodio[2], Lawrence H. Moulton[3], Dilruba Nasrin[5], Uma Onwuchekwa[2], Allison M. Shaffer[1], Samba O. Sow[2]**, Karen L. Kotloff[1,5]**

[1]Department of Pediatrics, Center for Vaccine Development and Global Health, University of Maryland School of Medicine, Baltimore.

[2]Centre pour le Développement des Vaccins, Bamako, Mali.

[3]Department of International Health, Johns Hopkins Bloomberg School of Public Health, Baltimore, MD, USA.

[4]Department of Obstetrics and Gynecology, Columbia University School of Medicine, New York, NY, USA.

[5]Department of Medicine, Center for Vaccine Development and Global Health, University of Maryland School of Medicine, Baltimore

*Amanda J. Driscoll and Fadima C. Haidara contributed equally to this work.

**Karen L. Kotloff and Samba O. Sow contributed equally to this work.

Corresponding Author:

Amanda J. Driscoll, MHS PhD

Center for Vaccine Development and Global Health

University of Maryland, Baltimore

685 W. Baltimore St.

Baltimore, MD 21201

Telephone (410) 706-5328

Email: adriscoll@som.umaryland.edu

Abstract

Introduction In high mortality settings, prophylactic azithromycin has been shown to improve birthweight and gestational age at birth when administered antenatally, to reduce the incidence of

For peer review only - http://bmjopen.bmj.com/site/about/guidelines.xhtml

neonatal infections when administered intrapartum, and to improve survival when administered in infancy. Questions remain regarding whether azithromycin can prevent stillbirths, and regarding the optimal strategy for the delivery of azithromycin to pregnant women and their infants.

Methods and analysis *Sauver avec l'Azithromycine en Traitant les Femmes Enceintes et les Enfants* (SANTE) is a 2x2 factorial, individually randomized, placebo-controlled, double-masked trial in rural Mali. The primary aims are: (1A) to assess the efficacy of antenatal and intrapartum azithromycin on a composite outcome of stillbirths and infant mortality through 6-12 months, and (1B) to assess the efficacy of azithromycin administered concurrently with the first and third doses of Pentavalent vaccines (Penta-1/3) on infant mortality through 6-12 months. Pregnant participants (n=49,600) and their infants are randomized 1:1:1:1 to one of four treatment arms: (1) mother and infant receive azithromycin, (2) mother and infant receive placebo, (3) mother receives azithromycin and infant receives placebo, or (4) mother receives placebo and infant receives azithromycin. Pregnant participants receive three single 2g doses: two antepartum and one intrapartum. Infants receive a single 20 mg/kg dose at the Penta-1 and 3 visits. An additional cohort of 12,000 infants is recruited at the Penta-1 visit and randomized 1:1 to receive azithromycin or placebo at the same timepoints. The results of SANTE will inform strategic decision-making regarding the efficacy of combined antenatal and infant azithromycin administration using routine health care delivery platforms

Ethics and dissemination This trial was approved by the Institutional Review Board at the University of Maryland School of Medicine (Protocol #HP-00084242) and the Faculté de Médecine et d'Odonto-Stomatologie in Mali. The findings of this trial will be published in open access peer-reviewed journals.

Trial registration number ClinicalTrials.gov number: NCT03909737; pre-results

https://clinicaltrials.gov/ct2/show/NCT03909737

Keywords: infectious diseases, epidemiology, perinatology, clinical trials

ARTICLE SUMMARY

STRENGTHS AND LIMITATIONS OF THIS STUDY

(5 short bullet points, no longer than one sentence each, that relate specifically to the methods)

- The trial is conducted in a low-resource setting with high rates of stillbirth and infant mortality
- The intervention is delivered through the public healthcare system, relying on the existing infrastructure for routine primary care
- The trial assesses multiple timepoints for the administration of azithromycin to prevent infant mortality (antenatal, intrapartum, and infant)

For peer review only - http://bmjopen.bmj.com/site/about/guidelines.xhtml

- The trial includes ancillary studies to assess antimicrobial resistance and to investigate potential mechanisms of action of azithromycin in subsets of participants
- The trial is unable to reliably determine individual causes of stillbirth and infant death

INTRODUCTION

Despite significant improvements in child survival over the past two decades, global rates of stillbirth and infant mortality remain unacceptably high. Of the 5.2 million under-five deaths in 2019, 3.9 million (75%) occurred within the first 12 months of life and 2.4 million (45%) within the first month.(1) More than half of global under-five deaths and 42% of the estimated 2 million global stillbirths occur in Sub-Saharan Africa, where approximately 60% of child mortality is estimated to be due to preventable infectious causes.(2, 3) Within the Child Health and Mortality Prevention Surveillance (CHAMPS) network, postmortem sampling identified at least one infectious condition in the causal chain leading to death in 53% of neonatal deaths and 17% of stillbirths (4). However, the causes of stillbirths in low- and middle-income countries (LMICs) are largely unexplained, owing to insufficient data and the failure to adopt a standardized classification system for perinatal cause of death.(5-7) . While malaria and syphilis are the most commonly recognized infectious causes of stillbirth, postmortem sampling in CHAMPS identified *Escherichia coli*/*Shigella*, *Streptococcus agalactia*, and *Enterococcus faecalis* as other potentially significant infectious causes.(4, 8)

An effective new strategy to prevent stillbirths and infant deaths that could be incorporated into routine primary healthcare services would be a major achievement in global health. One promising intervention to improve child survival is the prophylactic administration of the antibiotic azithromycin, a second-generation broad-spectrum macrolide that is used in mass drug administration (MDA) campaigns to control trachoma.(9) Current World Health Organization (WHO) guidelines recommend consideration of biannual MDA-azithromycin for infants aged 1-11 months to improve survival in high mortality settings.(10) These guidelines are based on data from three randomized controlled trials (RCTs), two of which found a statistically significant reduction in all-cause mortality among children randomized to receive azithromycin.(11-13) There are insufficient data to support global policy recommendations for prophylactic azithromycin in pregnancy, although it has also been shown to reduce the incidence of preterm birth and low birth weight when administered antenatally, and to prevent neonatal infections when administered intrapartum.(14, 15) Further evidence is needed to understand (1) whether antenatal and intrapartum azithromycin can prevent stillbirths and early infant deaths, (2) the optimal

For peer review only - http://bmjopen.bmj.com/site/about/guidelines.xhtml

method and timing of azithromycin administration in infancy to prevent mortality, (3) whether the survival benefit observed in two randomized MDA-azithromycin trials can be replicated in other settings.

Here, we describe the protocol for an individually randomized, placebo-controlled,2x2 factorial trial to determine (1) the effect of antenatal and intrapartum azithromycin on stillbirths and infant mortality and, (2) the effect of azithromycin on infant mortality when delivered with the first and third doses of diphtheria, tetanus, pertussis, hepatitis, and Hib-containing (i.e., pentavalent) vaccine. The trial will take place in Mali, a low-income West African country with high stillbirth, neonatal, and infant mortality.

For peer review only

METHODS AND ANALYSIS

Study Design, Objectives and Hypotheses

Sauver avec l'Azithromycine en Traitant les Femmes Enceintes et les Enfants (SANTE) is an individually randomized, placebo-controlled, double-masked trial. Using a 2x2 factorial design with four treatment arms, SANTE will simultaneously assess co-primary aim 1A: whether azithromycin administered at routine antenatal care (ANC) visits (second and/or third trimester) and at delivery reduces a composite outcome of stillbirth and infant mortality, and co-primary aim 1B: whether azithromycin administered to infants concurrently with the first and third doses of pentavalent vaccine (Penta-1 and Penta-3) reduces mortality from the time of the first dose through 6- 12 months of life. We will enroll 49,600 pregnant participants wo will be randomized to receive 2g azithromycin or placebo as a single oral dose twice during pregnancy and at delivery, for up to three total doses. Their infants are randomized to receive 20 mg/kg azithromycin or placebo as a single oral dose at their routine Penta-1 and Penta-3 vaccination visits (Figure 1). According to the national routine immunization schedule, these vaccination visits occur at approximately 6 and 14 weeks of age, respectively. Infants are followed through at least 6 months and up to 12 months of age. To increase statistical power for aim 1B, a separate cohort of 12,000 infants, the infant-only cohort, is recruited at the Penta-1 visit. Infants enrolled in the infant-only cohort are randomized, dosed, and followed in the same manner as those born to pregnant participants. Participant enrollment commenced in September 2020 and is expected to be complete by April 2023.

We hypothesize that the composite outcome of stillbirths and infant deaths will be lower in infants whose mothers were randomized to receive azithromycin in pregnancy compared to those randomized to receive placebo, and that mortality will be lower in infants who were randomized to receive

For peer review only - http://bmjopen.bmj.com/site/about/guidelines.xhtml

azithromycin at first and third pentavalent vaccination visits compared to those randomized to receive placebo.

The trial has been registered at ClinicalTrials.gov (NCT03909737). The WHO trial registration data set can be found online in the supplemental appendix, Table 1. Study Recruitment began in September 2020 and is anticipated to end by April 2023. All follow up will be complete within 12 months of enrolling the last pregnant participant.

Study Setting

SANTE is conducted by investigators at the Centre pour le Développement des Vaccins, Mali (CVD-Mali), an ongoing collaboration between the University of Maryland School of Medicine and the Malian Ministry of Health. For the past decade, much of northern and central Mali has been besieged by political instability and violence. The southwestern region of Sikasso, recently divided into the independent regions of Sikasso and Koutiala, had a reported infant mortality rate of 67 per 1000 live births in 2018.(16) It was selected as the study setting due to its high under-five mortality, moderate utilization of antenatal care and routine immunization coverage, and relative security (Supplemental Appendix, Table 2). Malaria is endemic in southwestern Mali, and standard of care for pregnant women is at least three doses of sulfadoxine-pyrimethamine (SP) intermittent preventive treatment (IPTp). Children aged 3-59 months receive a combination of monthly SP plus amodiaquine as seasonal malaria chemoprophylaxis (SMC) for four months during peak malaria transmission season (July-October). Each monthly SMC dose is comprised of one directly observed dose and two additional doses to be administered at home by the caregiver. In Sikasso, IPTp coverage with three or more doses is 26.8%, and 54% of age-eligible children receive one or more doses of SMC each year.(16) Within the Koutiala and Sikasso regions, the districts of Koutiala, Kignan, and Niena were selected for study operations. These districts represent areas with a high burden of pediatric illness and have populations of sufficient size to meet the study's enrollment requirement within the target timeframe.

Recruitment

Mali's decentralized health system operates at national, regional, district (*cercle*), health area (*aire de santé*), and community levels. Each district is served by a referral center (*Centre de Santé de Référence*, CSREF) and each *aire de santé* is served by one or more community health centers (*Centres de Santé Communautaire*, CSCOMs). SANTE study teams are embedded at all operational and accessible CSREFs and CSCOMs in the Koutiala, Kignan, and Niena districts. Many rural CSCOMs conduct regular community outreach operations (*stratégie avancée*), sending mobile units to remote health outposts to

For peer review only - http://bmjopen.bmj.com/site/about/guidelines.xhtml

provide ANC and immunization services. Study participants are recruited at antenatal or immunization visits at participating CSREFs and CSCOMs, or from remote health posts as part of the *stratégie avancée*.

Inclusion and Exclusion Criteria

Pregnant individuals seeking ANC are eligible to be enrolled if they have reached at least 14 weeks gestation and are not in active labor. If ultrasound dating is unavailable, participants are deemed eligible if the fundus can be palpated at two or more finger breadths above the pubic symphysis, a proxy measurement for a gestational age of at least 14 weeks.(17) Infants in the infant-only cohort are eligible to be enrolled if they are at least six weeks and fewer than 12 months of age and are presenting to a study facility to receive Penta-1 vaccine. Additional enrollment criteria are provided in Table 1.

Table 1. Inclusion and Exclusion Criteria

**Enrollment Criteria: Pregnant women and unborn infants enrolled in mother-infant cohort**	
**Inclusion Criteria**	**Exclusion Criteria**
• Participant is estimated to be ≥14 weeks gestation according to the study algorithm and is presenting at a study facility	• Participant is in active labor, as defined by study standard operating procedures
• Participant understands and can comply with planned study procedures, as determined by the study staff	• Participant has a known allergy to macrolide antibiotics
• Participant or legal authority has provided informed consent prior to initiation of any study procedures	• Participant has a condition that, in the opinion of the investigator, might compromise his or her well-being, or affect the ability of the participant to comply with study procedures
• Participant intends to reside in the study area until her newborn infant is at least 6 months old	• Participant is currently being treated with a macrolide antibiotic for a medical condition (this can be a temporary exclusion if the drug is later discontinued)
**Enrollment Criteria: Infants enrolled in the infant-only cohort**	
**Inclusion Criteria**	**Exclusion Criteria**
• Participant is presenting for Penta-1 vaccination at a study facility	• Participant has a known allergy to macrolide antibiotics
• Participant is at least 6 weeks of age, and fewer than 12 months of age	• Participant has a condition that, in the opinion of the investigator, might compromise his or her well-being, or affect the ability of the participant to comply with study procedures
• Participant's parent/guardian understands and can comply with the study procedures, as determined by the study staff	• Participant is currently being treated with a macrolide antibiotic for a medical condition

For peer review only - http://bmjopen.bmj.com/site/about/guidelines.xhtml

	(this can be a temporary exclusion if the drug is later discontinued)
• Participant's parent or guardian has provided informed consent prior to initiation of any study procedures	
• Participant's parent or guardian intends to reside in the study area until infant is at least 6 months old	

For peer review only

Randomization and masking

Treatment allocation is stratified by enrollment facility and randomized in permuted blocks of varying sizes (Supplemental Appendix). Participants are randomized 1:1:1:1 to one of the treatment assignments, with four possible assignments in the mother-infant cohort and two possible assignments in the infant-only cohort (**Figure 1**). Infants born to mothers enrolled in the mother-infant cohort are randomized when the mother is enrolled, i.e., before the infant is born. Infants born to multiple gestation pregnancies are randomized to the same treatment group.

Participants are randomized at enrollment using barcoded labels that are pre-printed with a sequential participant identification number and randomization assignment. The randomization assignment corresponds to a masked treatment bin, denoted by a letter of the alphabet, and pre-printed on every study medication bottle. Participants, field workers and study investigators are unaware of the link between the bin letters and the treatment assignments.

Intervention

Participants receive either azithromycin (Zithromax®) or placebo, both donated by the Pfizer Corporation. Both have the same appearance and color, but the placebo contains no active substance. Study medication is reconstituted at the time of dosing and administered under direct observation in oral suspension form.

Pregnant participants are dosed with 50mL of reconstituted liquid, equivalent to 2g of study medication. Infants are dosed 20 mg/kg up to a maximum of 1g, consistent with the recommended dosage for MDA.(10) Infant doses are automatically calculated by the electronic data collection form according to the infant's weight. Participants are observed for 30 minutes after receiving each dose. Participants who

For peer review only - http://bmjopen.bmj.com/site/about/guidelines.xhtml

have an allergic reaction to the study medication are given medical attention as appropriate and are retained in the study but do not receive additional doses.

Dosing timepoints are aligned with routine healthcare visits. A minimum 72-hour window is required between doses. Pregnant participants receive up to three single doses over the course of the study, comprised of 1-2 antenatal doses and a dose at delivery (**Table 2**).

Table 2. Study Schedule

**Timepoint**	**ANC visit ≥14 weeks gestation**	**Second ANC visit**	**Delivery**	**1 week after delivery**	**Penta-1 visit (approx. 6 weeks after delivery)**	**Penta-3 visit (approx. 14 weeks after delivery)**	**6 months after delivery**	**12 months after delivery[1]**
**Mother-Infant Cohort participants**								
Eligibility assessment, and informed consent	M							
Randomization	M/I							
Administration of study medication	M	M	M		I	I		
Demographic Questionnaire	M							
Medical history	M	M	M		M/I	I		
Labor and Delivery Questionnaire			M/I					
Weight measurement			I		I	I		
Vital Status Assessment		M	M/I	I	M/I	I	I	I
**Infant-only Cohort Participants**								
Eligibility Assessment, informed consent, and randomization					I			
Administration of study medication					I	I		
Demographic Questionnaire					I			
Medical history					I	I		
Weight measurement					I	I		
Vital Status					I	I	I	I

ANC= antenatal care
Penta-1 = First pentavalent vaccination
Penta-3= Third pentavalent vaccination
M=Mother participant; I=Infant participant
[1]Infants who reach 12 months of age before the end of follow up have a final vital status assessment at 12 months. All other infants have a final vital status assessment at the end of the study follow up period when they are between 6 and 12 months of age.

For peer review only - http://bmjopen.bmj.com/site/about/guidelines.xhtml

Ideally, pregnant participants are dosed in the second and third trimesters, but to accommodate individual variations in ANC timing, the first dose is administered at the enrollment ANC visit and the second dose at the next routine ANC visit or at delivery, whichever occurs sooner. The delivery dose is given during labor when possible but may be administered within 24 hours after delivery regardless of whether the infant is stillborn or liveborn. Infants are dosed when they present to a study facility to receive their first and third doses of pentavalent vaccine. Infants must be at least six weeks of age to receive the first dose of study medication.

Follow up and Primary Outcome measurements

The primary study outcomes are a composite of stillbirths and infant deaths for aim 1A, and infant deaths for aim 1B. Stillbirths are defined according to the WHO early stillbirth definition, i.e., the birth of a fetus with no signs of life at ≥22 weeks gestation, or the birth of a fetus with no signs of life weighing at least 500g.(18) Birth outcomes are recorded for every enrolled participant at the delivery visit. Participants who miss the delivery visit are followed up at least three times to ascertain the birth outcome.

Infant deaths are defined as any death of a liveborn infant in the first year of life, with infant vital status assessed at multiple timepoints (**Table 2**). All enrolled infants are followed through at least six months of age. Study follow up ends when the last enrolled infant reaches six months of age. At that point, all infants between six and 12 months of age will have a final vital status assessment. Infants who reach 12 months of age before the end of the trial will have a final vital status assessment at 12 months. Participants with a six-month vital status that is unknown after at least three contact attempts by the study team are considered lost to follow-up.

Secondary Outcome measurements

Secondary outcomes include low birth weight (<2500g), gestational age at birth (Supplemental Appendix, Table 3), neonatal death (0-<28 days), and a composite outcome of maternal hospitalizations and mortality through six weeks postpartum. The cost effectiveness of the interventions will also be estimated as a secondary aim.

Safety monitoring and adverse events

Azithromycin is considered safe for use in pregnancy and is recommended for the treatment of urethritis and cervicitis caused by *Chlamydia trachomatis* and *Neisseria gonorrhoeae.* (19) Increased risks of malformations have been reported in some observational studies following first trimester

For peer review only - http://bmjopen.bmj.com/site/about/guidelines.xhtml

exposure to azithromycin or to any macrolide antibiotic,(20-24) but not in others.(25-28) In a meta-analysis, azithromycin use (vs. alternative antibiotics) in the first trimester was weakly associated with gastrointestinal malformations.(29) In the second and third trimesters, macrolide exposure was associated with genital malformations, comprised mostly of hypospadias.(20) One case control study reported increased relative odds of miscarriage in the first 20 weeks of pregnancy among those prescribed azithromycin.(30) A limitation of these observational studies is the potential for confounding by indication. In SANTE, participants are enrolled after the first 14 weeks of pregnancy and study personnel are trained to identify and report major congenital malformations as serious adverse events (SAEs). Other events considered as SAEs include pregnancy losses before 22 weeks, maternal deaths, hospitalizations, and other significant or life-threatening illnesses. SAEs are reviewed by study physicians and assessed for relatedness to study medication.

In young children, the most common adverse reactions to azithromycin are gastrointestinal symptoms, reported in 1-4% of those treated.(10, 31) Cardiac toxicity is a potential concern, although the risk is not well-established in pediatric populations.(10) A randomized trial of 450 children aged six weeks to 59 months found no severe adverse events in azithromycin treated children, and only vomiting was reported more frequently in the azithromycin arm.(32) An association between treatment with erythromycin and infantile hypertrophic pyloric stenosis (IHPS) has been reported in the first two weeks of life, and to a lesser extent, in the third through fifth weeks of life.(33) The relationship between azithromycin and IHPS in infants less than six weeks of age is unclear.(34) In a recent RCT, one case of IHPS was reported among more than 10,000 azithromycin-treated infants fewer than six months of age.(35) The first dose of study medication in SANTE is administered at age 6 weeks or greater, although breastfed infants born to enrolled mothers could be exposed to low levels of azithromycin following the maternal delivery dose.(36) IHPS is monitored in SANTE as an adverse event of special interest and symptoms are solicited at all infant visits. An algorithm prompts physician review and assessment for relatedness, with any instances of probable or confirmed IHPS reported as SAEs (Supplemental Appendix, Figure 1).

To detect potential harmful associations between azithromycin and the SANTE study endpoints, an independent statistician analyzes stillbirths and infant deaths by treatment group at pre-specified intervals. The integrity of the trial is maintained by limiting these analyses to the harmful side of the sampling distribution of the hazard and risk ratio statistics. Results are reported to the data safety and monitoring board (DSMB).

For peer review only - http://bmjopen.bmj.com/site/about/guidelines.xhtml

Statistical Considerations

Sample size and power

The original power calculations for the primary aims were based on anticipated stillbirth and infant mortality rates in the Sikasso/Koutiala regions estimated from published data at the time the trial was being designed (**Table 3**). Analyzable endpoints were expected for at least 85% of enrolled participants. After the trial was initiated, observed stillbirth and infant mortality rates in the study population, pooled across blinded treatment arms, were used to update the sample size targets from 33,600 to 49,600 in the mother-infant cohort and from 8,500 to 12,000 in the infant-only cohort (**Supplemental Appendix, Table 4**). The resulting sample sizes are large, as infant mortality even in Mali is relatively rare, resulting in somewhat small absolute differences. For example, for Aim1B, with a baseline IMR of 9/1000, a 24.7% relative reduction means detecting an absolute reduction of less than 3 deaths per 1000.

Table 3. Sample size assumptions and minimum detectable relative reductions for the co-primary aims

	**Original**	**Revised**
**Target Sample size, mother-infant cohort**	33,600[1]	49,600
**Aim 1A. Effect of intervention on composite outcome of stillbirths and infant deaths from birth to 12 months**		
Baseline combined stillbirth and infant mortality rate	105/1000[2]	74/1000[2]
Minimum detectable relative mortality reduction, comparing azithromycin to placebo treatment arms		
80% power	9.5%	8.7%
90% power	10.9%	10.1%
**Aim 1B. Effect of intervention on infant deaths from first dose (approx. 6 weeks) to 12 months**		
Infant mortality rate	28/1000[3]	9/1000[3]
Minimum detectable relative mortality reduction, comparing azithromycin to placebo treatment arms		
80% power	18.6%	24.7%
90% power	21.4%	28.3%
With addition of infants from the infant-only cohort[4]		
80% power	16.6%	22.3%
90% power	19.1%	25.6%

[1]In the original sample size calculation, 15% anticipated loss to follow up (LTFU) meant that 28,560 infants were expected to have analyzable endpoints.

For peer review only - http://bmjopen.bmj.com/site/about/guidelines.xhtml

[2] Original assumed composite baseline stillbirth and infant mortality was 105 deaths per 1000 births, calculated from 33 stillbirths / 1000 births (37) and 74.8 infant deaths per 1000 live births (Sikasso data, Mali 2012 DHS report (38)), assuming that 6-11 month mortality data is available for 90% of infants and subtracting stillbirths from the live birth denominator).Revised observed composite stillbirth and infant mortality rate in the study population is 74 deaths per 1000 births.
[3]Original baseline infant mortality rate after 6 weeks of age was 28 per 1000 live births (Sikasso data, 2012 Mali DHS report) assuming that 6-11 month mortality data is available for 90% of infants; revised infant mortality rate after 6 weeks of age was updated to 9 deaths per 1000 live births based on observed mortality in the study population
[4]Original target sample size for infant-only cohort was 8,500, assuming 7,650 analyzable (10% LTFU); revised target sample size 12,000

For peer review only

Analysis of primary aims

The primary analysis for aim 1A will be according to the intention to treat (ITT) principle. All live born infants and all stillborn infants born at ≥22 weeks gestation, or weighing at least 500g, will be included, with infants classified according to the treatment assignment made at the time of the mother's enrollment. For aim 1B, the primary analysis will be according to modified ITT, beginning from the first assigned dose. Sensitivity analyses of the two aims will be modified as-treated. If a mother or infant receives at least one study dose of azithromycin, they will be classified as being in the corresponding azithromycin-receiving study arm. Percent lost-to-follow up will be compared across treatment arms for each intervention. Among those LTFU, baseline characteristics will be compared to assess whether loss is differential by intervention or by treatment arm. In the 2x2 factorial design, the primary analyses will fit a Cox proportional hazards regression model with two indicator variables for the two interventions, with censoring at the earliest of documented refusal for further participation, death, last study contact or recorded source of vital status, or attainment of 12 months of age. The study is not powered to detect a statistical interaction effect between the two interventions, but if it is important ($p<0.05$ or ratio of hazard ratios >1. 5 or <0.67), results for all four study arms also will be reported. For primary aim 1A, the analysis timeline will be calculated from delivery, with stillbirths assigned day 0.1. For primary aim 1B, the timeline will start at the receipt of the first infant dose. For a measure of overall public health impact on infant mortality, a supplementary analysis will be done with the timeline starting at the live birth.

Interim Analysis

There is one pre-specified formal interim analysis for efficacy planned for the maternal intervention, to take place when half of the expected number of stillbirths and infant deaths have occurred. This analysis

For peer review only - http://bmjopen.bmj.com/site/about/guidelines.xhtml

will be run with an interim Type I error of 0.007, which is expected to require a maternal intervention relative efficacy of at least 13%. Due to the complexity of this 2x2 factorial design, if this level were reached, the DSMB would consider all available data, and decide whether to stop randomization to one or both interventions.

Cost Effectiveness Analysis

We will conduct a cost-effectiveness analysis of maternal, infant, and combination azithromycin delivery, reflecting the arms of the trial. The economic cost of program implementation will be estimated by summing the market price of azithromycin with costs of drug storage and personnel time for administration. Health outcomes will include maternal and infant mortality as measured for each arm, converted into disability-adjusted life-years (DALYs) based on life tables for Mali. We will calculate incremental cost-effectiveness ratios compared to the status quo and for progressively expensive strategies.

Ancillary Studies

Ancillary studies will be carried out in subsets of participants. Longitudinal sampling of mother-infant pairs will measure antimicrobial resistance of *Streptococcus pneumoniae* and *Escherichia coli* isolates and identify genomic markers of resistance. The impact of co-administration of azithromycin with rotavirus vaccine will be assessed. Several ancillary studies are aimed at elucidating the mechanisms by which azithromycin might prevent stillbirths and infant deaths, including investigations of the impact of azithromycin on maternal and infant anthropometry, the vaginal microbiome, the maternal and infant gut microbiomes, biomarkers of environmental enteric dysfunction, and malaria in pregnancy and infancy.

Data Collection and Management

All study data are collected electronically on tablets using the open-source Tangerine data collection platform. The data collection platform is developed and maintained by RTI International, who serves as the data coordinating center (DCC). Data are collected offline, and tablets are regularly synced to a cloud-based server for central aggregation and processing. Data are managed collaboratively by the DCC and study teams.

Community Engagement and Consent

Community engagement was initiated in the study planning phase and will continue throughout the trial period. Engagement takes place through a series of meetings including local health center personnel,

For peer review only - http://bmjopen.bmj.com/site/about/guidelines.xhtml

religious and cultural leaders, and other community stakeholders at the regional and district levels. At each meeting, the investigators explain the trial procedures, the risks and benefits of participation, and other relevant information. A dynamic exchange ensues, and permission from the community leaders must be obtained to initiate and continue the trial.

Ethics and Dissemination

Written informed consent is obtained from the participant or their guardian. The study is approved by the ethics committees representing the University of Maryland, Baltimore, and the Faculté de Médecine et d'Odonto-Stomatologie in Mali. Results of the trial will be shared with the communities where participants were recruited, Mali's Ministry of Health, the WHO, and other stakeholders. A data sharing policy will be implemented. Results will be published in the peer-reviewed literature.

Study Oversight and Quality Assurance

A DSMB comprised of experts in pediatrics, obstetrics, biostatistics, and research ethics oversees patient safety and data collection for the trial. External monitoring visits are conducted quarterly. The study medication is analyzed on a routine basis by an independent laboratory.

Patient and Public Involvement

Local stakeholders in Mali will be involved in the research dissemination plans.

DISCUSSION

The SANTE trial is poised to address important knowledge gaps regarding the potential for azithromycin to improve pregnancy outcomes and infant survival in a high mortality setting. Unique attributes of SANTE compared to other RCTs of azithromycin in pregnancy include (1) a large sample size with statistical power to detect a reduction in stillbirths and infant mortality, (2) a combined antenatal and intrapartum intervention, and (3) the assessment of maternal azithromycin as a standalone randomized intervention rather than one given in combination with other antibiotics or as part of an iPTP regimen.

With antibacterial, antiprotozoal, antimalarial, immunomodulatory, and possibly antiviral properties, azithromycin delivered antenatally could prevent stillbirths and neonatal deaths by acting on causal or contributing factors including maternal infection, sterile or infection-related inflammation, and poor maternal nutritional status.(39-43) Support for these hypothesized mechanisms of action is provided by randomized trials showing a reduction in low birthweight and preterm birth following antenatal azithromycin.(44-46) In The Gambia, a single 2g dose of intrapartum azithromycin reduced bacterial carriage and the incidence of non-severe infection in neonates.(15, 47) Whether this finding translates

For peer review only - http://bmjopen.bmj.com/site/about/guidelines.xhtml

to reduced neonatal sepsis and death is being assessed in a follow up trial (NCT03199547). A separate multi-site trial will assess whether 2g intrapartum azithromycin prevents sepsis and death in neonates and their mothers (NCT03871491). We did not identify any published RCTs of azithromycin in pregnancy with stillbirth or infant death as the primary aim. Those that included a stillbirth outcome as a non-primary aim found no effect.(46, 48, 49) Three trials reported a reduction in neonatal (46, 50) or post-neonatal mortality (51) while others found no difference by mortality by treatment arm.(15, 44, 48, 52, 53)

Evidence of azithromycin's effect on child mortality comes from three large, randomized trials. The first reported a 49% reduction in all-cause mortality (95% CI 10, 71) among Ethiopian children 1-9 years residing in communities randomized to receive MDA-azithromycin compared to those in control communities.(11) The second, known as the MORDOR trial, found a 13.5% (95%CI 6.7, 19.8) reduction in mortality in children 1-59 months and a 24.9% (95% CI 10.6, 37.0) reduction in infants 1-5 months who resided in communities randomized to receive biannual MDA-azithromycin compared to placebo. (12) Although MORDOR was designed to detect a mortality effect across sites in three countries, a statistically significant benefit was only observed in Dosso, Niger, the site with the highest under-five mortality. To maximize the potential benefit of the infant intervention in SANTE, we situated the study in a region of Mali where the infant mortality rate is similar to that of the Dosso region.(54, 55) Targeted administration earlier in infancy, when the risk of death is greater, and the addition of a second dose within the first six months of life could potentially increase the survival benefit for infants in SANTE above that observed in MORDOR.

In contrast to the MDA-azithromycin trials, Chandramohan et al. reported no reduction in hospitalizations or mortality among children aged 3-59 months in Burkina Faso and Mali who were randomized at the household level to receive monthly SMC in combination with either azithromycin or placebo during the malaria transmission season.(13) One potential explanation for the difference in findings is that mortality reduction in the MORDOR sites, where routine SMC was not implemented, might have been due to azithromycin's antimalarial properties, and that there was no additional benefit of azithromycin beyond concurrent SMC in the Chandramohan trial. Children 3-59 months residing in the SANTE study area will be eligible to receive SMC during the malaria transmission season, but there are several reasons that SMC might be less influential in the SANTE trial. First, most infant deaths occur before three months of age. Second, only a subset of infants enrolled in SANTE will reach three months of age during the malaria season and thus be eligible for SMC. Third, only 54% of caregivers in Sikasso

For peer review only - http://bmjopen.bmj.com/site/about/guidelines.xhtml

reported that their age-eligible child received one or more SMC doses during the malaria season.(16) This suggests that SMC coverage among infants enrolled in SANTE will be considerably lower than in the Chandramohan trial, where it was administered to all participants as part of the study intervention.

Most recently, the NAITRE trial in Burkina Faso individually randomized infants at 8-27 days of age to receive a single oral dose of 20mg/kg azithromycin or placebo and found no reduction in mortality through six months of age.(35) Important differences between SANTE and NAITRE include different dosing schedules, higher baseline infant mortality in the SANTE study area, the inclusion of very remote populations in SANTE, and the inclusion of infants in the early neonatal period in SANTE.

The SANTE trial has two important limitations. The first is that ascertaining a reliable cause of death is not possible in the rural study setting. This limits inferences that can be made about differences in cause of death by treatment arm, although SANTE will conduct ancillary studies to understand possible mechanisms by which azithromycin might prevent mortality. Second, SANTE's individually randomized design will likely not achieve the interruption of pathogen transmission that might have occurred in MDA trials. One reason why a significant survival benefit for azithromycin has so far only been demonstrated in trials that randomized at the community level could be that the mechanism of protection requires high intervention coverage to interrupt pathogen transmission.(56)

Conclusion

SANTE is a large 2x2 factorial individually randomized placebo-controlled trial in a high mortality setting designed to generate evidence regarding the use of prophylactic azithromycin in pregnancy and early infancy to prevent stillbirths and infant deaths.

Acknowledgements

The authors would like to acknowledge the following individuals for their many contributions to this work:

Bill & Melinda Gates Foundation– Laura Lamberti

Data and Safety Monitoring Board – *Baylor College of Medicine* – Flor Muñoz-Rivas; *University of Washington School of Medicine* - Linda Eckert; *University of Mali, Bamako* – Allassane Dicko; *Faculté de Médicine Pharmacie et d'OdontoStomatologie* – Saïbou Maiga; *University of California at San Francisco* – Travis Porco

For peer review only - http://bmjopen.bmj.com/site/about/guidelines.xhtml

SANTE Study Group - *University of Maryland, Baltimore* –Wilbur Chen, Fleesie Hubbard, Yuanyuan Liang, Jacques Ravel, Ousmane S. Sow, Sharon Tennant, Mark Travassos; *Centre pour le Développement des Vaccins, Bamako, Mali* –Mamadou Diallo, Jane Juma, Awa Traore - *RTI International* - Matthew Finholt-Daniel, Emily Hadley, Jennifer J. Hemingway-Foday, Chris Kelley, Alice Litavecz, Elizabeth McClure, Melissa Page, David Plotner, RJ (Steinert) Corwin, Kevin Wilson; *PATH* – Robert Choy; *Institute for Health Metrics and Evaluation, University of Washington, Seattle, WA, United States* -Michael Arndt.

Author contributions

Contributors: KLK is the principal investigator of the trial. KLK conceived and designed the trial with substantial contributions from SOS, AJD, and LHM, in addition to FCH, ELD, and MDT. AJD, FCH, MDT, ELD, MCF, RLG, LHM, SOS and KLK wrote the protocol. LHM wrote the statistical analysis plan. AJD, FCH, MDT, ELD, OSS, TB, JAB, MK, DN, AMS, RLG, UO, SOS and KLK developed data collection materials. SOS and FCH will lead the implementation of the trial with contributions from AJD, KLK, MDT, OSS, TB, MK and UO. AJD drafted and revised the manuscript, with input from KLK. All authors critically reviewed and approved the final manuscript.

Funding statement

This work was supported by the Bill & Melinda Gates Foundation grant number 1196125. The funders played no role in the design, writing, or decision to publish the study protocol.

Ethics Approval

Ethical approval was obtained for the SANTE study from the Institutional Review Boards at the University of Maryland, Baltimore (Protocol # HP- 00084242) and the Faculté de Médecine et d'Odonto-Stomatologie in Mali.

Competing Interests

Dr. Moulton serves on the Data Safety and Monitoring Board for Pfizer COVID-19 and respiratory syncytial virus vaccine trials.

Figure Legend

Figure 1. SANTE study design. Flow diagram to illustrate randomization and treatment groups for the 2x2 factorial mother-infant cohort and the infant-only cohort. "Penta 1" and "Penta 3" correspond to the first and third doses of pentavalent vaccine (i.e., diphtheria, tetanus, pertussis, hepatitis, and Hib-containing vaccine).

References

1. Estimation UI-aGfCM. Levels and Trends in Child Mortality - Report 2020. Geneva, Switzerland; 2020.

For peer review only - http://bmjopen.bmj.com/site/about/guidelines.xhtml

2. Global, regional, national, and selected subnational levels of stillbirths, neonatal, infant, and under-5 mortality, 1980-2015: a systematic analysis for the Global Burden of Disease Study 2015. Lancet (London, England). 2016;388(10053):1725-74.
3. UN Inter-agency group for Child Mortality Estimation. A Neglected Tragedy: The global burden of stillbirths. Geneva; 2020.
4. Taylor AW, Blau DM, Bassat Q, Onyango D, Kotloff KL, Arifeen SE, et al. Initial findings from a novel population-based child mortality surveillance approach: a descriptive study. The Lancet Global health. 2020;8(7):e909-e19.
5. Reinebrant HE, Leisher SH, Coory M, Henry S, Wojcieszek AM, Gardener G, et al. Making stillbirths visible: a systematic review of globally reported causes of stillbirth. BJOG : an international journal of obstetrics and gynaecology. 2018;125(2):212-24.
6. Goldenberg RL, Harrison MS, McClure EM. Stillbirths: The Hidden Birth Asphyxia - US and Global Perspectives. Clinics in perinatology. 2016;43(3):439-53.
7. Goldenberg RL, Muhe L, Saleem S, Dhaded S, Goudar SS, Patterson J, et al. Criteria for assigning cause of death for stillbirths and neonatal deaths in research studies in low-middle income countries. The journal of maternal-fetal & neonatal medicine : the official journal of the European Association of Perinatal Medicine, the Federation of Asia and Oceania Perinatal Societies, the International Society of Perinatal Obstet. 2018:1-9.
8. McClure EM, Silver RM, Kim J, Ahmed I, Kallapur M, Ghanchi N, et al. Maternal infection and stillbirth: a review. The journal of maternal-fetal & neonatal medicine : the official journal of the European Association of Perinatal Medicine, the Federation of Asia and Oceania Perinatal Societies, the International Society of Perinatal Obstet. 2020:1-9.
9. Taylor HR, Burton MJ, Haddad D, West S, Wright H. Trachoma. Lancet (London, England). 2014;384(9960):2142-52.
10. Organization WH. WHO guideline on mass drug administration of azithromycin to children under five years of age to promote child survival 2020.
11. Porco TC, Gebre T, Ayele B, House J, Keenan J, Zhou Z, et al. Effect of mass distribution of azithromycin for trachoma control on overall mortality in Ethiopian children: a randomized trial. Jama. 2009;302(9):962-8.
12. Keenan JD, Bailey RL, West SK, Arzika AM, Hart J, Weaver J, et al. Azithromycin to Reduce Childhood Mortality in Sub-Saharan Africa. The New England journal of medicine. 2018;378(17):1583-92.
13. Chandramohan D, Dicko A, Zongo I, Sagara I, Cairns M, Kuepfer I, et al. Effect of Adding Azithromycin to Seasonal Malaria Chemoprevention. The New England journal of medicine. 2019;380(23):2197-206.
14. Hume-Nixon M, Quach A, Reyburn R, Nguyen C, Steer A, Russell F. A Systematic Review and meta-analysis of the effect of administration of azithromycin during pregnancy on perinatal and neonatal outcomes. EClinicalMedicine. 2021;40:101123.
15. Oluwalana C, Camara B, Bottomley C, Goodier S, Bojang A, Kampmann B, et al. Azithromycin in Labor Lowers Clinical Infections in Mothers and Newborns: A Double-Blind Trial. Pediatrics. 2017;139(2).
16. Institut National de la Statistique - INSTAT CdPedSSS-DSePdlFCS-D-PeI. Enquête Démographique et de Santé au Mali 2018. Bamako, Mali et Rockville, Maryland, USA : INSTAT, CPS/SS-DS-PF et ICF.; 2018.
17. Krelin Naidu KLF. Gestational Age Assessment [Updated 2021 Aug 2]. Treasure Island (FL): StatPearls Publishing; Jan 2022.
18. Organization WH. Making every baby count: audit and review of stillbirths and neonatal deaths at facility. Geneva; 2016.
19. Prevention CfDCa. Sexually Transmitted Infections Treatment Guidelines, 2021. 2021. Contract No.: 4.

For peer review only - http://bmjopen.bmj.com/site/about/guidelines.xhtml

20. Fan H, Gilbert R, O'Callaghan F, Li L. Associations between macrolide antibiotics prescribing during pregnancy and adverse child outcomes in the UK: population based cohort study. BMJ (Clinical research ed). 2020;368:m331.
21. Bojang A, Baines SL, Camara B, Guerillot R, Donovan L, Marqués RS, et al. Impact of Intrapartum Oral Azithromycin on the Acquired Macrolide Resistome of Infants' Nasopharynx: A Randomized Controlled Trial. Clinical infectious diseases : an official publication of the Infectious Diseases Society of America. 2020;71(12):3222-5.
22. Muanda FT, Sheehy O, Bérard A. Use of antibiotics during pregnancy and the risk of major congenital malformations: a population based cohort study. Br J Clin Pharmacol. 2017;83(11):2557-71.
23. Leke AZ, Dolk H, Loane M, Casson K, Nelen V, Barišić I, et al. Macrolide and lincosamide antibiotic exposure in the first trimester of pregnancy and risk of congenital anomaly: A European case-control study. Reprod Toxicol. 2021;100:101-8.
24. Mallah N, Tohidinik HR, Etminan M, Figueiras A, Takkouche B. Prenatal Exposure to Macrolides and Risk of Congenital Malformations: A Meta-Analysis. Drug Saf. 2020;43(3):211-21.
25. Andersson NW, Olsen RH, Andersen JT. Association between use of macrolides in pregnancy and risk of major birth defects: nationwide, register based cohort study. BMJ (Clinical research ed). 2021;372:n107.
26. Cooper WO, Hernandez-Diaz S, Arbogast PG, Dudley JA, Dyer SM, Gideon PS, et al. Antibiotics potentially used in response to bioterrorism and the risk of major congenital malformations. Paediatric and perinatal epidemiology. 2009;23(1):18-28.
27. Bérard A, Sheehy O, Zhao JP, Nordeng H. Use of macrolides during pregnancy and the risk of birth defects: a population-based study. Pharmacoepidemiol Drug Saf. 2015;24(12):1241-8.
28. Damkier P, Brønniche LMS, Korch-Frandsen JFB, Broe A. In utero exposure to antibiotics and risk of congenital malformations: a population-based study. American journal of obstetrics and gynecology. 2019;221(6):648.e1-.e15.
29. Fan H, Li L, Wijlaars L, Gilbert RE. Associations between use of macrolide antibiotics during pregnancy and adverse child outcomes: A systematic review and meta-analysis. PloS one. 2019;14(2):e0212212.
30. Muanda FT, Sheehy O, Bérard A. Use of antibiotics during pregnancy and risk of spontaneous abortion. Cmaj. 2017;189(17):E625-e33.
31. Zeng L, Xu P, Choonara I, Bo Z, Pan X, Li W, et al. Safety of azithromycin in pediatrics: a systematic review and meta-analysis. Eur J Clin Pharmacol. 2020;76(12):1709-21.
32. Sié A, Dah C, Bountogo M, Ouattara M, Nebie E, Coulibaly B, et al. Adverse Events and Clinic Visits following a Single Dose of Oral Azithromycin among Preschool Children: A Randomized Placebo-Controlled Trial. The American journal of tropical medicine and hygiene. 2020;104(3):1137-41.
33. Eberly MD, Eide MB, Thompson JL, Nylund CM. Azithromycin in early infancy and pyloric stenosis. Pediatrics. 2015;135(3):483-8.
34. Smith C, Egunsola O, Choonara I, Kotecha S, Jacqz-Aigrain E, Sammons H. Use and safety of azithromycin in neonates: a systematic review. BMJ open. 2015;5(12):e008194.
35. Oldenburg CE, Sié A, Bountogo M, Zakane A, Compaoré G, Ouedraogo T, et al. Neonatal Azithromycin Administration for Prevention of Infant Mortality. NEJM Evidence. 2022;1(4):EVIDoa2100054.
36. Azithromycin. Drugs and Lactation Database (LactMed). Bethesda (MD): National Library of Medicine (US); 2006.
37. Blencowe H, Cousens S, Jassir FB, Say L, Chou D, Mathers C, et al. National, regional, and worldwide estimates of stillbirth rates in 2015, with trends from 2000: a systematic analysis. The Lancet Global health. 2016;4(2):e98-e108.
38. DHS. Mali DHS, 2012-2013 Final Report. Rockville, MD: The DHS Program, USAID; 2012-2013.

For peer review only - http://bmjopen.bmj.com/site/about/guidelines.xhtml

39. Khoshnood S, Shirani M, Dalir A, Moradi M, Haddadi MH, Sadeghifard N, et al. Antiviral effects of azithromycin: A narrative review. Biomed Pharmacother. 2022;147:112682.
40. Kricker JA, Page CP, Gardarsson FR, Baldursson O, Gudjonsson T, Parnham MJ. Nonantimicrobial Actions of Macrolides: Overview and Perspectives for Future Development. Pharmacol Rev. 2021;73(4):233-62.
41. McClure EM, Goldenberg RL. Infection and stillbirth. Seminars in fetal & neonatal medicine. 2009;14(4):182-9.
42. Baker BC, Heazell AEP, Sibley C, Wright R, Bischof H, Beards F, et al. Hypoxia and oxidative stress induce sterile placental inflammation in vitro. Scientific reports. 2021;11(1):7281.
43. McClure EM, Saleem S, Pasha O, Goldenberg RL. Stillbirth in developing countries: a review of causes, risk factors and prevention strategies. The journal of maternal-fetal & neonatal medicine : the official journal of the European Association of Perinatal Medicine, the Federation of Asia and Oceania Perinatal Societies, the International Society of Perinatal Obstet. 2009;22(3):183-90.
44. Unger HW, Ome-Kaius M, Wangnapi RA, Umbers AJ, Hanieh S, Suen CS, et al. Sulphadoxine-pyrimethamine plus azithromycin for the prevention of low birthweight in Papua New Guinea: a randomised controlled trial. BMC medicine. 2015;13:9.
45. Luntamo M, Kulmala T, Mbewe B, Cheung YB, Maleta K, Ashorn P. Effect of repeated treatment of pregnant women with sulfadoxine-pyrimethamine and azithromycin on preterm delivery in Malawi: a randomized controlled trial. The American journal of tropical medicine and hygiene. 2010;83(6):1212-20.
46. Gray RH, Wabwire-Mangen F, Kigozi G, Sewankambo NK, Serwadda D, Moulton LH, et al. Randomized trial of presumptive sexually transmitted disease therapy during pregnancy in Rakai, Uganda. American journal of obstetrics and gynecology. 2001;185(5):1209-17.
47. Roca A, Oluwalana C, Bojang A, Camara B, Kampmann B, Bailey R, et al. Oral azithromycin given during labour decreases bacterial carriage in the mothers and their offspring: a double-blind randomized trial. Clinical microbiology and infection : the official publication of the European Society of Clinical Microbiology and Infectious Diseases. 2016;22(6):565.e1-9.
48. Kimani J, Phiri K, Kamiza S, Duparc S, Ayoub A, Rojo R, et al. Efficacy and Safety of Azithromycin-Chloroquine versus Sulfadoxine-Pyrimethamine for Intermittent Preventive Treatment of Plasmodium falciparum Malaria Infection in Pregnant Women in Africa: An Open-Label, Randomized Trial. PloS one. 2016;11(6):e0157045.
49. Moore BR, Benjamin JM, Tobe R, Ome-Kaius M, Yadi G, Kasian B, et al. A Randomized Open-Label Evaluation of the Antimalarial Prophylactic Efficacy of Azithromycin-Piperaquine versus Sulfadoxine-Pyrimethamine in Pregnant Papua New Guinean Women. Antimicrobial agents and chemotherapy. 2019;63(10):e00302-19.
50. Hendrixson DT, Smith K, Lasowski P, Callaghan-Gillespie M, Weber J, Papathakis P, et al. A novel intervention combining supplementary food and infection control measures to improve birth outcomes in undernourished pregnant women in Sierra Leone: A randomized, controlled clinical effectiveness trial. PLoS medicine. 2021;18(9):e1003618.
51. Hallamaa L, Cheung YB, Maleta K, Luntamo M, Ashorn U, Gladstone M, et al. Child Health Outcomes After Presumptive Infection Treatment in Pregnant Women: A Randomized Trial. Pediatrics. 2018;141(3).
52. Tita AT, Szychowski JM, Boggess K, Saade G, Longo S, Clark E, et al. Adjunctive Azithromycin Prophylaxis for Cesarean Delivery. The New England journal of medicine. 2016;375(13):1231-41.
53. Kalilani L, Mofolo I, Chaponda M, Rogerson SJ, Alker AP, Kwiek JJ, et al. A randomized controlled pilot trial of azithromycin or artesunate added to sulfadoxine-pyrimethamine as treatment for malaria in pregnant women. PloS one. 2007;2(11):e1166.

For peer review only - http://bmjopen.bmj.com/site/about/guidelines.xhtml

54. International INdlS-INaI. Enquête Démographique et de Santé et à Indicateurs Multiples du Niger 2012. Calverton, Maryland, USA; 2013.
55. DHS. Mali DHS, Final Report 2012-2013. Rockville, MD: The DHS Program, USAID; 2012-2013.
56. O'Brien KS, Oldenburg CE. MDA and trial designs to evaluate the impact of azithromycin on child mortality. The Lancet Global health. 2022;10(2):e183.

For peer review only

For peer review only - http://bmjopen.bmj.com/site/about/guidelines.xhtml

SANTE Protocol– FIGURE – August 8, 2022

Figure 1.

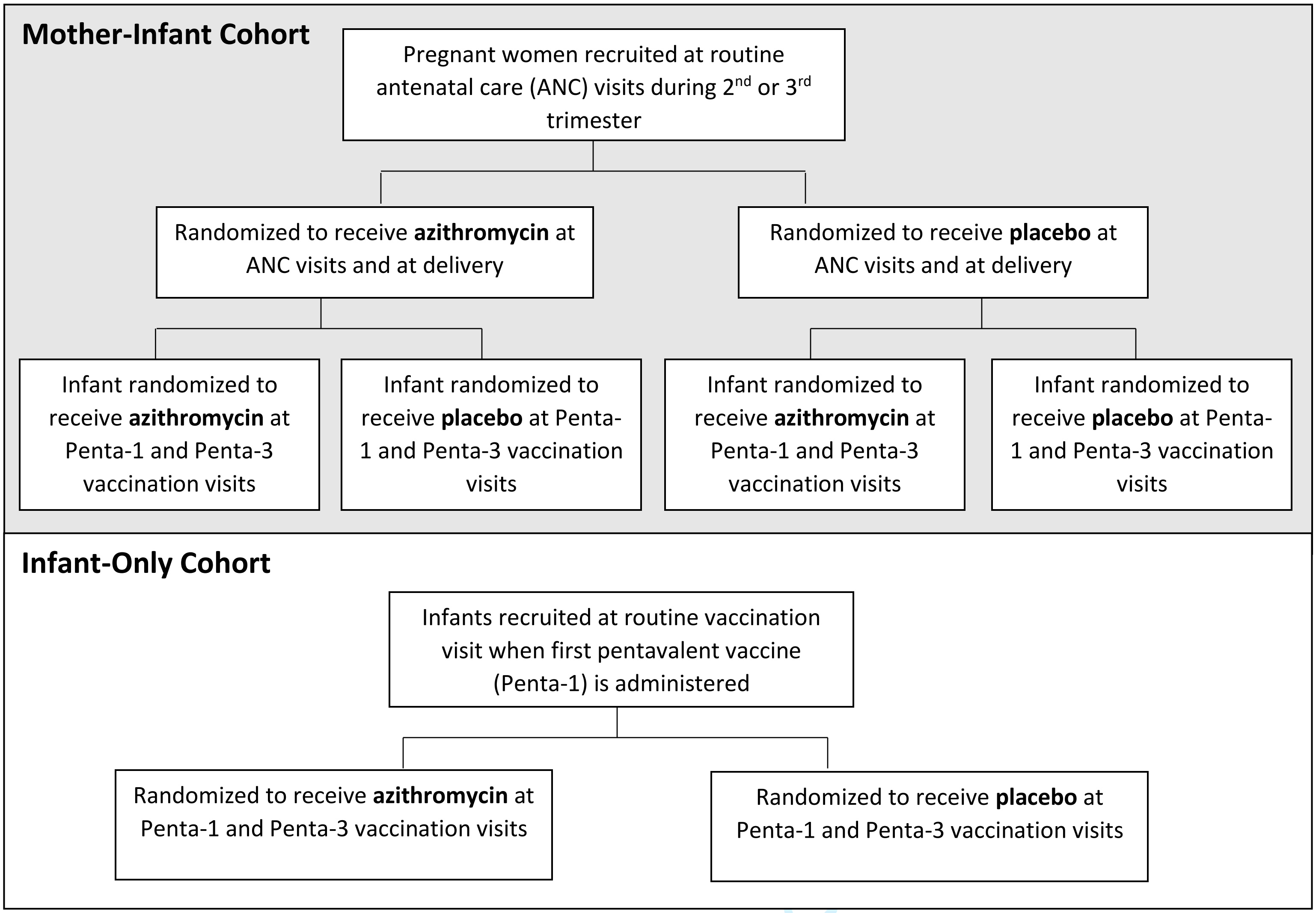

Supplemental Online Appendix.

Supplemental Table 1. World Health Organization Trial Registration Data Set

**Primary Registry and Trial Identifying Number**	ClinicalTrials.gov; NCT03909737
**Date of Registration in Primary Registry**	April 10, 2019
**Secondary Identifying Numbers**	
**Source(s) of Monetary or Material Support**	Bill & Melinda Gates Foundation
**Primary Sponsor**	University of Maryland, Baltimore
**Secondary Sponsor(s)**	n/a
**Contact for Public Queries**	Amanda Driscoll PhD<br>adriscoll@som.umaryland.edu
**Contact for Scientific Queries**	Karen Kotloff MD<br>Karen Kotloff kkotloff@som.umaryland.edu
**Public Title**	The SANTE trial
**Scientific Title**	Sauver avec l'Azithromycine en Traitant les Femmes Enceintes et les Enfants / Save with Azithromycin by Treating Pregnant Women and Children (SANTE)
**Countries of Recruitment**	Mali
**Health conditions or problems studied**	The burden of stillbirths and infant mortality remains high in low-income settings. Prophylactic azithromycin delivered in pregnancy and in infancy has shown promise as a potential intervention to improve birth outcomes and infant survival.
**Interventions**	Pregnant women: a single 2g dose of oral azithromycin (or placebo) delivered in the second and third trimesters of pregnancy, and at delivery<br>Infants: a single 20mg/kg dose of oral azithromycin (or placebo) delivered concurrently with the first and third doses of diphtheria, tetanus, pertussis containing vaccine.
**Key Inclusion and Inclusion Criteria**	At enrollment, pregnant women must be ≥14 weeks gestation and must not be in active labor. Infant participants must be ≥6 weeks of age at enrollment and/or the time of the first dose. Participants must plan to reside in the study area for at least 6 months after the infant is born. Participants who are being treated with azithromycin or another macrolide antibiotic for a medical condition are excluded from enrollment.
**Study Type**	Individually randomized 2x2 factorial placebo-controlled trial
**Date of First Enrollment**	September 2020
**Sample size**	49,600 mother-infant pairs, plus an additional 12,000 infants

For peer review only - http://bmjopen.bmj.com/site/about/guidelines.xhtml

**Recruitment Status**	Recruiting
**Primary Outcome(s)**	Primary outcome 1A: a composite of stillbirths and infant mortality within the first 6-12 months of life<br>Primary outcome 1B: infant mortality between the time of first does through 6-12 months of life
**Key Secondary Outcomes**	Low birth weight (<2500g), neonatal death (0-<28 days), and a composite outcome of maternal hospitalizations and mortality through six weeks postpartum. The cost effectiveness of the interventions will also be estimated as a secondary aim.
**Ethics Review**	This trial was approved by the Institutional Review Board at the University of Maryland School of Medicine (Protocol #HP-00084242) and the Faculté de Médecine et d'Odonto-Stomatologie in Mali
**Completion Date**	n/a
**Summary Results**	n/a
**IPD Sharing Statement**	Access to trial IPD may be provided to qualified researchers upon request and following review and approval of a research proposal and execution of a Data Sharing Agreement (DSA).

Supplemental Table 2. Characteristics of the Study Settings

	Sikasso Region (SANTE study area)	Comparator Indicators		
		Mali, national level indicators	African Region	Global
Under-five mortality rate per 1000 live births	111[1]	101[1]	76[2]	38[2]
Infant mortality rate per 1000 live births	67[1]	54[1]	52[2]	28[2]
Neonatal mortality rate per 1000 live births	42[1]	33[1]	27[2]	17[2]

For peer review only - http://bmjopen.bmj.com/site/about/guidelines.xhtml

Perinatal deaths per 1000 births (stillbirths plus early neonatal deaths w/in 7 days)[2]	45[3]	38[3]		
Stillbirths per 1000 births	7[3]	12[3] / 19.7[4]	21.7[4]	13.9[4]
Proportion of pregnancies where antenatal care was received by a trained provider	76.1%[5]	79.5%[5]		
Proportion of pregnant women that received 3+ doses of IPTp	26.8%[6]	28.3%[6]	35%[7]	
Proportion of births that were facility-based	71.0%[5]	66.8%[5]		
Proportion of births attended by a skilled provider	70.8%[5]	67.3%[5]		
Proportion of children 12-35 months of age who received DTP-1	85.7%[8]	82.1%[8]		
Proportion of children 12-35 months of age who received DTP-3	69.4%[8]	70.7%[8]		

[1] From Mali 2018 DHS Report, Tables 8.1 and 8.2

[2] 2019 data, from Levels & Trends in Child Mortality. Report 2020. Estimates developed by the UN Inter-agency Group for Child Mortality Estimation.

[3] From Mali 2018 DHS Report, Table 8.4. Stillbirths calculated as the difference between perinatal and early neonatal deaths, and defined as fetal deaths occurring at 7 months or more of pregnancy

[4] 2019 estimates from: Global, regional, and national estimates and trends in stillbirths from 2000 to 2019: a systematic assessment. 2021. Lancet 398:772-785. Stillbirth defined as fetal death at 28 weeks gestation or more.

[5] From Mali 2018 DHS Report, Tables 9.1, 9.5 and 9.6 – Trained providers defined as doctors, nurses, and midwives.

[6] IPTp= Intermittent preventive treatment for malaria during pregnancy. From Mali 2018 DHS Report, Table 12.9

[7] 2019 estimate from World Malaria Report, 2021. World Health Organization. https://www.who.int/teams/global-malaria-programme/reports/world-malaria-report-2021

[8] DTP=Diphtheria, tetanus and pertussis containing vaccine. From Mali 2018 DHS Report, Table 10.3

For peer review only - http://bmjopen.bmj.com/site/about/guidelines.xhtml

For peer review only

Supplemental Table 3. Gestational Age Algorithm*

**For participants with only fundal health (FH) measurement available:**	
If FH measurement ≥16 cm or ≥35 cm	Assume cm FH = weeks gestational age
If FH measurement <16 cm	Assume GA= 14-15 weeks
If FH measurement >35 cm	Assume GA>35 weeks
**For participants with date of last menstrual period (LMP date) available:**	
If no FH or ultrasound dating (US) available	Use LMP date
If no US available and difference between LMP date and FH is +/- 14 days	Use LMP date
If no US available and difference between LMP date and FH is > 14 days	Use FH
If US is available:	
Earliest US at <9 weeks	
Within +/- 5 days of LMP date	Use LMP date
> 5 days different from LMP date	Use US
Earliest US at 9 to <16 weeks	
Within +/- 7 days of LMP date	Use LMP date
> 7 days different from LMP date	Use US
Earliest US at 16 to <22 weeks	
Within +/- 10 days of LMP date	Use LMP date
> 10 days different from LMP date	Use US
Earliest US at 22 to <28 weeks	
Within +/- 14 days of LMP date	Use LMP date
> 14 days different from LMP date	Use US
Earliest US at ≥28 weeks	
Within +/- 21 days of LMP date	Use LMP date
> 21 days different from LMP date	Use US
**For participants without FH, LMP date, or US**	Estimated GA is missing

*Adapted with permission from the Azithromycin-Prevention in Labor Use Study (A-PLUS) investigators https://clinicaltrials.gov/ct2/show/NCT03871491

For peer review only

Supplemental Figure 1. Infantile Hypertrophic Pyloric Stenosis Reporting Algorithm

Asked at each infant visit: Has the infant had forceful vomiting after feeding for 2 or more days, with vomit the color of breastmilk?

↓

If yes, complete Adverse Event of Special Interest Form, shown below

**1.** Date of notification:	___ ___/___ ___ ___/___ ___ ___ ___(dd/MMM/yyyy)
**2.** Date symptoms began	___ ___ ___ ___ Year (*enter 9999 if unknown*)<br>___ ___ ___ Month (*enter XXX if unknown*)<br>___ ___ Day (*enter 99 if unknown*)
**3.** Are the symptoms ongoing?	☐ YES (*skip to 4*)<br>☐ NO<br>☐ Unknown (*skip to 4*)
**3a**. (If NO to Q3) End date of symptoms	___ ___ ___ ___ Year (*enter 9999 if unknown*)<br>___ ___ ___ Month (*enter XXX if unknown*)<br>___ ___ Day (*enter 99 if unknown*)
**4.** Date of the last dose of study drug:*	___ ___/___ ___ ___/___ ___ ___ ___<br>(dd/MMM/yyyy)
**Event information**	
**5.** Age of the infant participant*	___ ___ weeks
**6.** Sex of the infant participant*	☐ Male ☐ Female ☐ Indeterminate
**7.** How many consecutive days did the vomiting last?	________ Days
**8.** Does the infant participant seem to be hungry most of the day? (*select one option*)	☐ YES ☐ NO ☐ Unknown
**9.** Has the infant participant had diarrhea?	☐ YES ☐ NO ☐ Unknown
**10.** Did the parent/caregiver seek medical care for this vomiting episode? (*If NO, refer for a medical evaluation if the symptoms are ongoing*)	☐ YES ☐ NO ☐ Unknown
**10a**. (If YES to Q10) What was the diagnosis? (*select one option*)	☐ Pyloric stenosis<br>☐ Gastroenteritis<br>☐ Other, specify: __________<br>☐ Unknown
**10b**. (If YES to Q10) How was the diagnosis made? (*choose all options that apply*)	☐ Clinical exam (feeling a mass)<br>☐ Ultrasound<br>☐ Surgery<br>☐ Other, specify ________________<br>☐ Unknown
**10c.** (If YES to Q10) What care was provided? (*choose all options that apply*)	☐ Medicine<br>☐ Surgery<br>☐ None<br>☐ Unknown
**11.** Pyloric stenosis diagnosis*	☐ Possible (Forceful vomiting of 2 days or more color of breastmilk)<br>☐ Probable (Possible, with palpitation of a mass) (*Complete SAE form*)<br>☐ Definite (Sonogram, ultrasound, or surgical diagnosis) (*Complete SAE form*)

For peer review only - http://bmjopen.bmj.com/site/about/guidelines.xhtml

Randomization Procedures

Randomization for the main study will be performed via stratified permuted-block design.

For the mother-infant cohort:

- Randomization of the mother-infant pairs will be stratified by facility and will occur at the enrollment ANC visit
- Since the table of randomization numbers and corresponding treatment assignments will be generated in advance, extra randomization numbers will be generated for each (facility) as we do not know in advance how many women will be randomized within each stratum
- Within each stratum, randomization to treatment groups A1, A2, B1 and B2 will be performed using a permuted block design with randomly varying block sizes of 8 and 16
    - A total of 4,992 treatment assignments will be generated in each stratum and there will be a total of 416 blocks – 208 of size 8 and 208 of size 16
- Within each block, 25% of the treatment assignments will be allocated to each treatment (A1, A2, B1, B2)
- Mother and infant treatment assignments will each be allocated two bins; thus, there will be four bins (two for active and two for placebo) for mother treatments and four bins (two for active and two for placebo) for infant treatments
- Bins will be labeled with letters, as in the following example, for a total of 4*2 (8) different randomization assignments:
    - **Mother (active) – bins A and B;**
    - **Mother (placebo) – bins C and D;**
    - **Infant (active) – bins E and F;**
    - **Infant (placebo) – bins G and H;**
- Bins will be assigned to each mother/infant treatment assignment in equal proportion. For example, half of the mother (active) randomization assignments will be bin A and the other half will be bin B.
- Each randomization assignment will be associated with one bin.

For the infant-only cohort:

- Randomization will be stratified by facility
- Since the table of randomization numbers and corresponding treatment assignments will be generated in advance, extra randomization numbers will be generated for each stratum as we do not know in advance how many infants will be randomized within each stratum
- Within each stratum, randomization to treatment groups 1 and 2 will be performed using a permuted block design with randomly varying block sizes of 4 and 8
    - A total of 1800 treatment assignments will be generated in each stratum and there will be a total of 300 blocks –150 of size 4 and 150 of size 8

For peer review only - http://bmjopen.bmj.com/site/about/guidelines.xhtml

- Within each block 50% of the treatment assignments will be allocated to each treatment (1 and 2)
- Infant treatment assignments will each be allocated two bins; thus, there will be four bins (two for active and two for placebo)
- Bins will be labeled with letters, as follows, for a total of 2*2 (4) different randomization assignments:
    - **Infant (active) – bins E and F**
    - **Infant (placebo) – bins G and H**

Bins will be assigned to each infant treatment assignment in equal proportion

For peer review only

Supplemental Table 4. Protocol Modifications

Protocol version	Date	Major modifications
1.0	14Jan2019	Original submission
2.0	13Mar2019	-Added descriptions of ancillary studies<br>-Added pilot acceptability testing<br>-Added procedures for obtaining consent electronically<br>-Increased overall enrollment number
3.0	19Oct2019	-No major modifications; administrative clarifications only
4.0	19Nov2019	-No major modifications; administrative clarifications only
5.0	20Mar2020	-Gestational age requirement increased from 12 weeks to 14 weeks<br>-Added requirement for infants to be minimum 6 weeks of age for enrollment in infant-only cohort<br>-Removed pilot acceptability testing<br>-Clarified description of consenting procedures
6.0	12Mar2021	-Clarified infant dosing procedures to allow study medication dosing despite pentavalent vaccination stockouts, or to allow dosing if the infant has received vaccine at a non-study facility<br>-Excluded stillbirths from SAE reporting; added harm analysis for stillbirths<br>-Added ancillary studies
7.0	24Jun2021	-Allowed contacts between formal visits to assess health and vital status<br>-Exclude infant deaths from SAE reporting; added harm analysis for infant deaths
8.0	15Sep2021	No major changes, administrative clarifications
9.0	19May2022	Collection of nasopharyngeal swabs for ancillary studies changed to nasal swabs
10	26Oct2022	-Removal of urban mother-infant cohort<br>-Removal of ancillary studies that will not be enrolled<br>-Updated sample size calculations and target sample sizes

		-Excluded unplanned caesarean sections and obstetric emergencies from SAE reporting; added harm analysis for caesarean sections and obstetric emergencies

For peer review only

For peer review only - http://bmjopen.bmj.com/site/about/guidelines.xhtml

Reporting checklist for protocol of a clinical trial.

Based on the SPIRIT guidelines.

Instructions to authors

Complete this checklist by entering the page numbers from your manuscript where readers will find each of the items listed below.

Your article may not currently address all the items on the checklist. Please modify your text to include the missing information. If you are certain that an item does not apply, please write "n/a" and provide a short explanation.

Upload your completed checklist as an extra file when you submit to a journal.

In your methods section, say that you used the SPIRITreporting guidelines, and cite them as:

Chan A-W, Tetzlaff JM, Gøtzsche PC, Altman DG, Mann H, Berlin J, Dickersin K, Hróbjartsson A, Schulz KF, Parulekar WR, Krleža-Jerić K, Laupacis A, Moher D. SPIRIT 2013 Explanation and Elaboration: Guidance for protocols of clinical trials. BMJ. 2013;346:e7586

		Reporting Item	Page Number
**Administrative information**			
Title	#1	Descriptive title identifying the study design, population, interventions, and, if applicable, trial acronym	1

For peer review only

Trial registration	#2a	Trial identifier and registry name. If not yet registered, name of intended registry	1
Trial registration: data set	#2b	All items from the World Health Organization Trial Registration Data Set	S1-2
Protocol version	#3	Date and version identifier	n/a
Funding	#4	Sources and types of financial, material, and other support	13
Roles and responsibilities: contributorship	#5a	Names, affiliations, and roles of protocol contributors	1,13
Roles and responsibilities: sponsor contact information	#5b	Name and contact information for the trial sponsor	n/a
Roles and responsibilities: sponsor and funder	#5c	Role of study sponsor and funders, if any, in study design; collection, management, analysis, and interpretation of data; writing of the report; and the decision to submit the report for publication, including whether they will have ultimate authority over any of these activities	13
Roles and responsibilities: committees	#5d	Composition, roles, and responsibilities of the coordinating centre, steering committee, endpoint adjudication committee, data management team, and	11

For peer review only

		other individuals or groups overseeing the trial, if applicable (see Item 21a for data monitoring committee)	
**Introduction**			
Background and rationale	#6a	Description of research question and justification for undertaking the trial, including summary of relevant studies (published and unpublished) examining benefits and harms for each intervention	3, 8
Background and rationale: choice of comparators	#6b	Explanation for choice of comparators	3
Objectives	#7	Specific objectives or hypotheses	3
Trial design	#8	Description of trial design including type of trial (eg, parallel group, crossover, factorial, single group), allocation ratio, and framework (eg, superiority, equivalence, non-inferiority, exploratory)	3-4
**Methods: Participants, interventions, and outcomes**			
Study setting	#9	Description of study settings (eg, community clinic, academic hospital) and list of countries where data will be collected. Reference to where list of study sites can be obtained	4

For peer review only

Eligibility criteria	#10	Inclusion and exclusion criteria for participants. If applicable, eligibility criteria for study centres and individuals who will perform the interventions (eg, surgeons, psychotherapists)	4-6
Interventions: description	#11a	Interventions for each group with sufficient detail to allow replication, including how and when they will be administered	6
Interventions: modifications	#11b	Criteria for discontinuing or modifying allocated interventions for a given trial participant (eg, drug dose change in response to harms, participant request, or improving / worsening disease)	6
Interventions: adherance	#11c	Strategies to improve adherence to intervention protocols, and any procedures for monitoring adherence (eg, drug tablet return; laboratory tests)	7
Interventions: concomitant care	#11d	Relevant concomitant care and interventions that are permitted or prohibited during the trial	n/a
Outcomes	#12	Primary, secondary, and other outcomes, including the specific measurement variable (eg, systolic blood pressure), analysis metric (eg, change from baseline, final value, time to event), method of aggregation (eg, median, proportion), and time point for each outcome. Explanation of the clinical relevance of chosen efficacy and harm outcomes is strongly recommended	7-8

For peer review only

Participant timeline	#13	Time schedule of enrolment, interventions (including any run-ins and washouts), assessments, and visits for participants. A schematic diagram is highly recommended (see Figure)	6-7
Sample size	#14	Estimated number of participants needed to achieve study objectives and how it was determined, including clinical and statistical assumptions supporting any sample size calculations	8-9
Recruitment	#15	Strategies for achieving adequate participant enrolment to reach target sample size	4
**Methods: Assignment of interventions (for controlled trials)**			
Allocation: sequence generation	#16a	Method of generating the allocation sequence (eg, computer-generated random numbers), and list of any factors for stratification. To reduce predictability of a random sequence, details of any planned restriction (eg, blocking) should be provided in a separate document that is unavailable to those who enrol participants or assign interventions	5-6
Allocation concealment mechanism	#16b	Mechanism of implementing the allocation sequence (eg, central telephone; sequentially numbered, opaque,	5-6

For peer review only

		sealed envelopes), describing any steps to conceal the sequence until interventions are assigned	
Allocation: implementation	#16c	Who will generate the allocation sequence, who will enrol participants, and who will assign participants to interventions	5-6
Blinding (masking)	#17a	Who will be blinded after assignment to interventions (eg, trial participants, care providers, outcome assessors, data analysts), and how	5-6
Blinding (masking): emergency unblinding	#17b	If blinded, circumstances under which unblinding is permissible, and procedure for revealing a participant's allocated intervention during the trial	n/a
**Methods: Data collection, management, and analysis**			
Data collection plan	#18a	Plans for assessment and collection of outcome, baseline, and other trial data, including any related processes to promote data quality (eg, duplicate measurements, training of assessors) and a description of study instruments (eg, questionnaires, laboratory tests) along with their reliability and validity, if known. Reference to where data collection forms can be found, if not in the protocol	10

For peer review only

Data collection plan: retention	#18b	Plans to promote participant retention and complete follow-up, including list of any outcome data to be collected for participants who discontinue or deviate from intervention protocols	7
Data management	#19	Plans for data entry, coding, security, and storage, including any related processes to promote data quality (eg, double data entry; range checks for data values). Reference to where details of data management procedures can be found, if not in the protocol	10
Statistics: outcomes	#20a	Statistical methods for analysing primary and secondary outcomes. Reference to where other details of the statistical analysis plan can be found, if not in the protocol	8-10
Statistics: additional analyses	#20b	Methods for any additional analyses (eg, subgroup and adjusted analyses)	8-10
Statistics: analysis population and missing data	#20c	Definition of analysis population relating to protocol non-adherence (eg, as randomised analysis), and any statistical methods to handle missing data (eg, multiple imputation)	8-10
**Methods: Monitoring**			
Data monitoring: formal committee	#21a	Composition of data monitoring committee (DMC); summary of its role and reporting structure; statement of whether it is independent from the sponsor and competing interests; and reference to where further	11

For peer review only

		details about its charter can be found, if not in the protocol. Alternatively, an explanation of why a DMC is not needed	
Data monitoring: interim analysis	#21b	Description of any interim analyses and stopping guidelines, including who will have access to these interim results and make the final decision to terminate the trial	10
Harms	#22	Plans for collecting, assessing, reporting, and managing solicited and spontaneously reported adverse events and other unintended effects of trial interventions or trial conduct	8
Auditing	#23	Frequency and procedures for auditing trial conduct, if any, and whether the process will be independent from investigators and the sponsor	11
**Ethics and dissemination**			
Research ethics approval	#24	Plans for seeking research ethics committee / institutional review board (REC / IRB) approval	11
Protocol amendments	#25	Plans for communicating important protocol modifications (eg, changes to eligibility criteria, outcomes, analyses) to relevant parties (eg, investigators, REC / IRBs, trial participants, trial registries, journals, regulators)	n/a

For peer review only

Consent or assent	#26a	Who will obtain informed consent or assent from potential trial participants or authorised surrogates, and how (see Item 32)	11
Consent or assent: ancillary studies	#26b	Additional consent provisions for collection and use of participant data and biological specimens in ancillary studies, if applicable	n/a
Confidentiality	#27	How personal information about potential and enrolled participants will be collected, shared, and maintained in order to protect confidentiality before, during, and after the trial	n/a
Declaration of interests	#28	Financial and other competing interests for principal investigators for the overall trial and each study site	13
Data access	#29	Statement of who will have access to the final trial dataset, and disclosure of contractual agreements that limit such access for investigators	S2
Ancillary and post trial care	#30	Provisions, if any, for ancillary and post-trial care, and for compensation to those who suffer harm from trial participation	n/a
Dissemination policy: trial results	#31a	Plans for investigators and sponsor to communicate trial results to participants, healthcare professionals, the public, and other relevant groups (eg, via publication, reporting in results databases, or other data sharing arrangements), including any publication restrictions	11

For peer review only

Dissemination policy: authorship	#31b	Authorship eligibility guidelines and any intended use of professional writers	n/a
Dissemination policy: reproducible research	#31c	Plans, if any, for granting public access to the full protocol, participant-level dataset, and statistical code	S2

Appendices

Informed consent materials	#32	Model consent form and other related documentation given to participants and authorised surrogates	n/a
Biological specimens	#33	Plans for collection, laboratory evaluation, and storage of biological specimens for genetic or molecular analysis in the current trial and for future use in ancillary studies, if applicable	n/a

The SPIRIT Explanation and Elaboration paper is distributed under the terms of the Creative Commons Attribution License CC-BY-NC. This checklist was completed on 17. August 2022 using https://www.goodreports.org/, a tool made by the EQUATOR Network in collaboration with Penelope.ai

For peer review only
